# High-throughput materials screening algorithm based on first-principles density functional theory and artificial neural network for high-entropy alloys

**DOI:** 10.1038/s41598-022-21209-0

**Published:** 2022-10-05

**Authors:** Meena Rittiruam, Jakapob Noppakhun, Sorawee Setasuban, Nuttanon Aumnongpho, Attachai Sriwattana, Suphawich Boonchuay, Tinnakorn Saelee, Chanthip Wangphon, Annop Ektarawong, Patchanee Chammingkwan, Toshiaki Taniike, Supareak Praserthdam, Piyasan Praserthdam

**Affiliations:** 1 High-Performance Computing Unit (CECC-HCU), Center of Excellence on Catalysis and Catalytic Reaction Engineering (CECC), Chulalongkorn University, Bangkok, 10330 Thailand; 2 Center of Excellence on Catalysis and Catalytic Reaction Engineering (CECC), Chulalongkorn University, Bangkok, 10330 Thailand; 3 Rittiruam Research Group, Chulalongkorn University, Bangkok, 10330 Thailand; 4 Saelee Research Group, Chulalongkorn University, Bangkok, 10330 Thailand; 5 Extreme Conditions Physics Research Laboratory and Center of Excellence in Physics of Energy Materials (CE:PEM), Department of Physics, Faculty of Science, Chulalongkorn University, Bangkok, 10330 Thailand; 6 Chula Intelligent and Complex Systems, Faculty of Science, Chulalongkorn University, Bangkok, 10330 Thailand; 7 Graduate School of Advanced Science and Technology, Japan Advanced Institute of Science and Technology, 1-1 Asahidai, Nomi, Ishikawa 923-1292 Japan

**Keywords:** Density functional theory, Computational science, Scientific data, Chemical engineering

## Abstract

This work introduced the high-throughput phase prediction of PtPd-based high-entropy alloys via the algorithm based on a combined Korringa-Kohn-Rostoker coherent potential approximation (KKR-CPA) and artificial neural network (ANN) technique. As the first step, the KKR-CPA was employed to generate 2,720 data of formation energy and lattice parameters in the framework of the first-principles density functional theory. Following the data generation, 15 features were selected and verified for all HEA systems in each phase (FCC and BCC) via ANN. The algorithm exhibited high accuracy for all four prediction models on 36,556 data from 9139 HEA systems with 137,085 features, verified by R^2^ closed to unity and the mean relative error (MRE) within 5%. From this dataset comprising 5002 and 4137 systems of FCC and BCC phases, it can be realized based on the highest tendency of HEA phase formation that (1) Sc, Co, Cu, Zn, Y, Ru, Cd, Os, Ir, Hg, Al, Si, P, As, and Tl favor FCC phase, (2) Hf, Ga, In, Sn, Pb, and Bi favor BCC phase, and (3) Ti, V, Cr, Mn, Fe, Ni, Zr, Nb, Mo, Tc, Rh, Ag, Ta, W, Re, Au, Ge, and Sb can be found in both FCC and BCC phases with comparable tendency, where all predictions are in good agreement with the data from the literature. Thus, the combination of KKR-CPA and ANN can reduce the computational cost for the screening of PtPd-based HEA and accurately predict the structure, i.e., FCC, BCC, etc.

## Introduction

High-entropy alloys (HEAs) are classified by configurational entropy of mixing ($\Delta S$)^1^, in which the criteria are $\Delta S$ ≥ 1.36R and $\Delta S$ ≥ 1.50R for tetra and penta-metallic alloys^2^, respectively. This material has been employed in various applications due to its promising properties, especially catalytic^3–8^ and mechanical properties^9–12^. Nowadays, discovering new formulae of HEA via experimental techniques requires a high cost of chemicals and characterization^13^, where the phase and atomic composition are challenges for HEA materials^14^. Hence, the prediction of the possible phase formation based on computational techniques plays a crucial role in reducing HEA screening costs. It was demonstrated that a system with high $\Delta S$ in a multi-component alloy tended to form a single-phase HEA, implying that the system would be less likely to segregate^15^.

During these several years, machine learning (ML) techniques have been employed to predict structural properties and discover unknown materials^16^. However, a large amount of data is required for ML to predict such properties accurately. This will be the main obstacle if one would like to screen the materials based on experimental techniques. Complementing the experimental data if it is not enough; one should employ the first-principles techniques, e.g., density functional theory (DFT), to help generating enough training data for accurate ML prediction^17,18^. This coupling of ML and DFT can tremendously reduce the cost of materials discovery compared to the complete experimental materials screening, where only the expensive screening via high-throughput experimentation would be enough for ML^19^. Therefore, various researchers employed the ML-based method to explore new HEA in recent years, as Kaufmann and Vecchio^20^ reported, which employed the random forest technique to predict the single and multiple phases of binary, ternary, quaternary, and quinary alloys. They found that all predicted results agreed well with the validation from experimental data and the CALPHAD program. Moreover, Jin et al*.*^21^ exhibit the coupled DFT-ML technique to screen the phases of multi-component alloys. The training dataset of binary alloys was generated by DFT random alloy method, known as the Korringa-Kohn-Rostoker coherent potential approximation (KKR-CPA). As a result, their prediction accuracy was up to 80.56% for multi-component alloys and, interestingly, up to 84.20% for HEA materials^21^. Apart from such an algorithm, numerous techniques, i.e., the gradient boosting model, trained with 1,807 datasets, demonstrated high accuracy of 96.41% for predicting single-phase and non-single-phase refractory HEAs (RHEAs)^22^. Other methods also exhibit high accuracy prediction, e.g., a combined ML and CALPHAD technique, an artificial neural network technique (ANN) coupled with experimental data^9,10,12,23–28^. In addition to phase formation of HEAs, machine learning was recently employed to predict the mechanical properties of HEA bulk materials, including microhardness^10,27^, yield strength^12,23^, dislocation density^12^, elastic modulus^29^, Young’s modulus^30^, hardness^11,31^, and elastic constant^32^. These shed light on the ML-based high-throughput screening of HEA materials.

As in the aspect of the use of HEA as catalytic materials, the PtPd-based HEAs are among high potential candidates that can be utilized in CO_2_ and CO reduction reaction^7,8^, oxygen evolution reaction^33^, oxygen reduction reaction^34^, and hydrogen evolution reaction^35^. However, from the literature, it was found that most experimental works involving PtPd-based HEAs focused mainly on the report of limited formulae of HEA in terms of novel synthesis and characterization techniques^26,36–43^. Therefore, a gigantic set of possible HEA formulae is required to understand and utilize their promising properties fully. Hence, a firm protocol for screening HEA properties, such as their phase and electronic properties, must be studied and established. The phases and structural properties should be the first screening stage, in which bulk HEAs’ thermodynamic stability can be extracted through the formation energy (${\Delta E}_{f}$) at the ground-state configuration at 0 K^1^. Regarding such calculations, Miedema’s scheme model^44^ is one of the simple and fast methods used to obtain ${\Delta E}_{f}$, where the experimental technique must be used to obtain the individual energy for a monometallic component first. Yet, apart from the energy term, the structural information of a specific phase of alloys or compounds cannot be determined. To overcome such a problem, the periodic DFT was employed to determine both the ${\Delta E}_{f}$ and the structural information of a system is reported being successfully utilized in binary systems^45^. Nevertheless, to design the HEA, many possible atomic configurations have to be guessed and verified through thermodynamically optimized structures. The routine calculation via DFT alone would be intensive, although all information can be obtained. Also, suppose a combined DFT-ML technique is to be used for HEA discovery. In that case a huge configurational space must be generated to accurately determine the most favorable atomic configuration at a given operating temperature, pressure, and chemical composition. Thus, to reduce the computational cost, even more, we propose the combined KKR-CPA with ML technique for a rapid and low-cost HEA materials screening^46–49^. This method was proven by Jin et al*.*^21^ to be a successful tool for the property prediction of multi-metallic alloys. So far, such a method has not been applied to HEA screening. The KKR method employed is based on Green’s function method of multiple scattering theory to calculate the Green’s function of a system without knowing its eigenvalue of DFT^50–52^. Whereas the CPA method solves the problem of atomic configuration through the effective medium with the weighted average of Green’s function.

**Figure 1:**
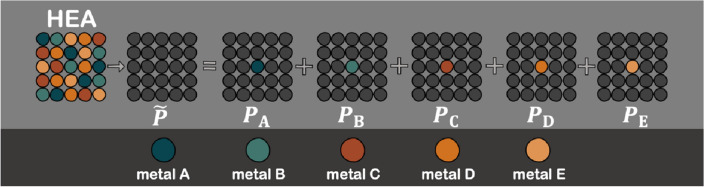
The coherent potential approximation (CPA) for the penta-metallic high-entropy alloy (HEA) recreated from Tian et al.^53^.

Accordingly, the CPA method is the most suitable for the multi-component system, such as the penta-metallic HEA comprising five metals: A, B, C, D, and E, as illustrated in Fig. 1. The CPA assumes the potential of each element (${P}_{\mathrm{A}}$, ${P}_{\mathrm{B}}$, ${P}_{\mathrm{C}}$, ${P}_{\mathrm{D}}$,${P}_{\mathrm{E}}$) without the effect of the local environment, where the atomic component is placed in the system in the effective medium ($\widetilde{P}$)^53^. $\widetilde{P}$ is determined selfconsistently with including all single site scattering events due to the respective atoms placed in the effective medium. It was previously reported that the KKR-CPA results were comparable to those obtained from a combined DFT and SQS (special quasi-random supercell) technique^54^. The validation of the KKR-CPA method for multi-component alloys is extensively explained in Sect. 1 of the Supporting Information based on the Akai-KKR software. Tables S1 and S2 demonstrate that the Akai-KKR package can indicate the stable phase for FCC and BCC alloys with a good agreement with available literature^54–65^.

**Figure 2:**
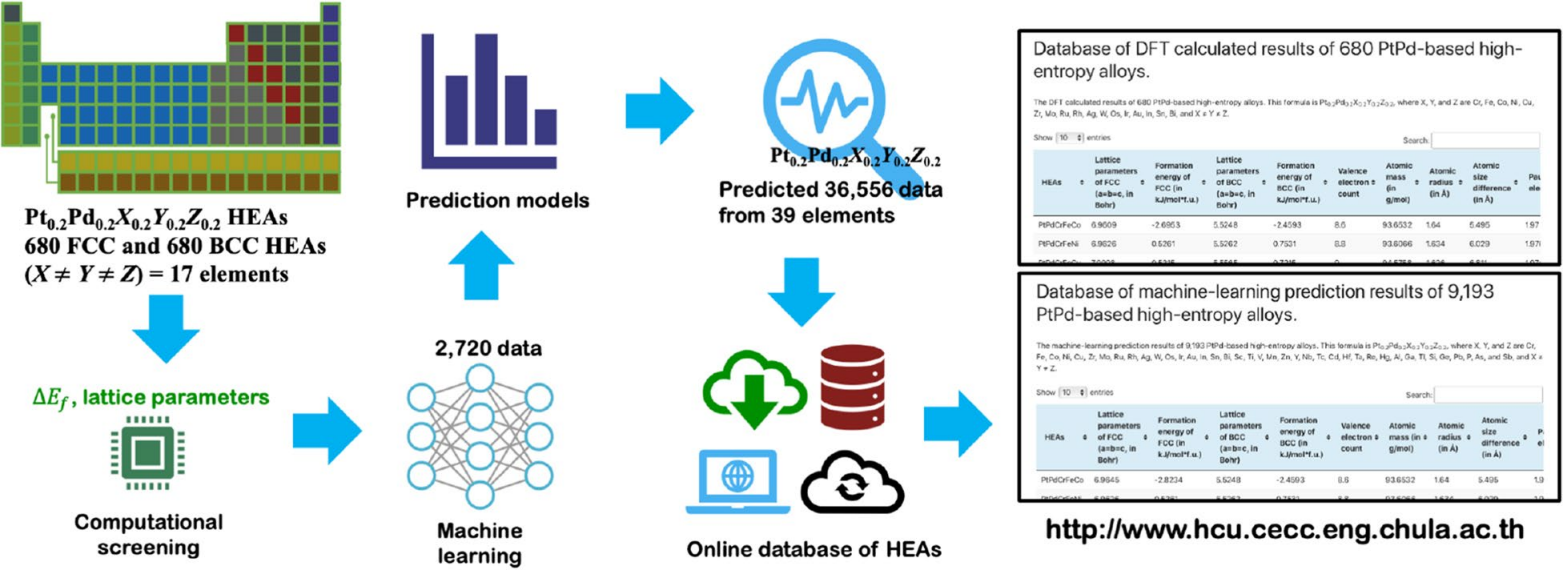
Our workflow based on first-principles density functional theory (DFT) and machine learning (ML) for the prediction of lattice parameter (*a*) and formation energy (${\Delta H}_{f}$) of HEA systems involving the utilization of our public database at http://www.hcu.cecc.eng.chula.ac.th/hea-database.

This work presents the ML prediction on the thermodynamically stable PtPd-based HEAs via ${\Delta E}_{f}$. The protocol is illustrated in Fig. 2. HEA formulae in focus are the Pt_0.2_Pd_0.2_*X*_0.2_*Y*_0.2_*Z*_0.2_ with *X*, *Y*, and *Z* being the components from a pool of 39 elements, i.e., Sc, Ti, V, Cr, Mn, Fe, Co, Ni, Cu, Zn, Y, Zr, Nb, Mo, Tc, Ru, Rh, Ag, Cd, Hf, Ta, W, Re, Os, Ir, Au, Hg, Al, Si, P, Ga, Ge, As, In, Sn, Sb, Tl, Pb, and Bi, where *X* $\ne$ *Y* $\ne$ *Z*. The training and test datasets of lattice parameters (*a*) and ${\Delta E}_{f}$ are obtained from the calculated results of 680 FCC and BCC HEA systems, constructed from 17 out of 39 elements mentioned above. The details of the DFT calculation and ML prediction model can be found in Sect. 2 of the Supporting Information. Consequently, the *a*(FCC), *a*(BCC), ${\Delta E}_{f}$(FCC), and ${\Delta E}_{f}$(BCC) of 9,139 HEAs (39 elements) are predicted via machine learning. The relative stability between FCC and BCC for a given HEA is obtained from the formation energy evaluated with respect to their constituent elements. Besides, 36,556 predicted data of 9139 HEAs with a total of 137,085 features are published on our public online database, as shown in Fig. 2. Finally, the screening rules for FCC and BCC-phase PtPd-based HEA are discussed and proposed.

## Theoretical methods

### DFT calculation

The ${\Delta E}_{f}$ of PtPd*-*based HEAs can be calculated as Eq. (1).

1 $${\Delta E}_{f}={E}_{\mathrm{total}}^{\mathrm{HEA}}\left(\mathrm{PtPd}XYZ\right)-\sum_{i}({c}_{i}{E}_{\mathrm{total}}^{\mathrm{element}}),$$

where ${E}_{\mathrm{total}}^{\mathrm{HEA}}\left(\mathrm{PtPd}XYZ\right)$ is the total energy for the HEA system, and ${E}_{\mathrm{total}}^{\mathrm{element}}$ is the total energy for each element calculated from the natural form, e.g., FCC Pt, BCC Mn, HCP Co, etc., The ${c}_{i}$ is the element concentration.

The total energies and lattice parameters of FCC and BCC PtPd*XYZ* HEAs were calculated using the Green’s function method implemented in Akai-KKR (Machikaneyama) package^51,52^. The KKR-CPA was employed for calculating the electronic structure of the random alloy system comprising *n* components. The crystal potential was approximated by using the muffin-tin potential with the atomic-sphere approximation. The Perdew–Wang-91 generalized gradient approximation (GGA91)^66^ was used for an exchange–correlation functional. The self-consistent calculation was performed using the criteria of 8 × 8 × 8k-point mesh in the first Brillouin-zone. The electron density was calculated from the imaginary part of the Green’s function evaluated on the complex energy contour whose width is 1.0 Ry from the Fermi energy. The iteration was performed until the difference between input and output potential becomes 10^–6^. The maximum angular momentum for the expansion of Green’s function was set to 2. The scalar relativistic approximation (SRA) was used for the relativistic treatment.

### Machine learning

**Figure 3:**
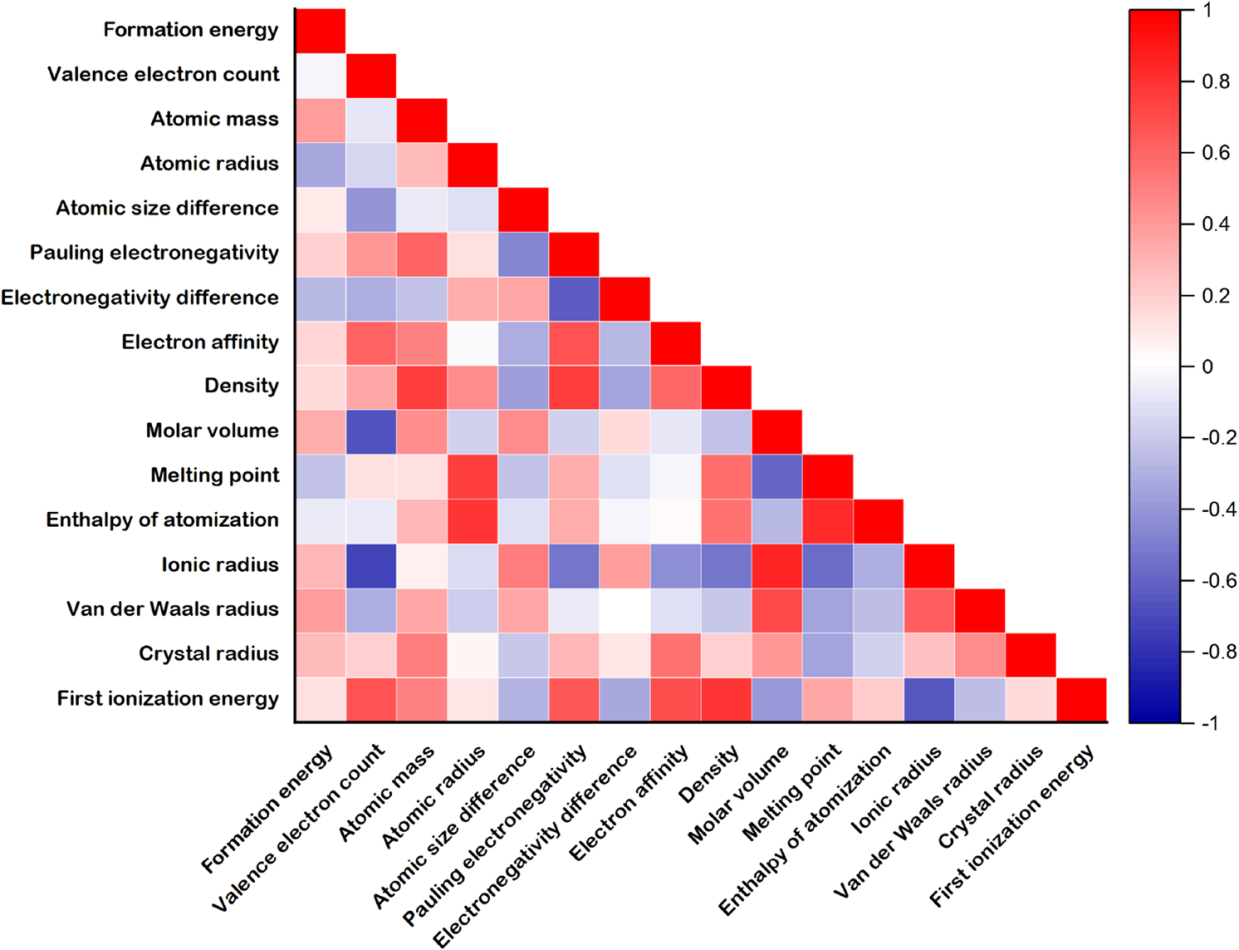
Matrix representation of Pearson correlation coefficient of features. The formation energy corresponds to ${\Delta E}_{f}$(FCC) of HEAs.

The 2,720 data of *a* and ${\Delta H}_{f}$ were collected from the DFT calculation of FCC and BCC PtPd*XYZ* HEAs (*X* $\ne$ *Y* $\ne$ *Z* = Cr, Fe, Co, Ni, Cu, Zr, Mo, Ru, Rh, Ag, W, Os, Ir, Au, In, Sn, Bi) including 680 data of each FCC and BCC HEA for making each ML prediction model viz., *a*(FCC), *a*(BCC), ${\Delta H}_{f}$(FCC), and ${\Delta H}_{f}$(BCC). Fifteen features from the physical and chemical properties of each element, including valence electron count (VEC), atomic mass ($M$), atomic radius (${r}_{atomic}$), atomic size difference ($\delta$), Pauling electronegativity ($\chi$), electronegativity difference ($\Delta \chi$), electron affinity ($EA$), density ($\rho$), molar volume (${V}_{mol}$), melting point (${T}_{mel}$), enthalpy of atomization ${\Delta H}_{f,\mathrm{atom}}$, ionic radius (${r}_{ionic}$), Van der Waals radius (${r}_{\mathrm{VdW}}$), crystal radius (${r}_{\mathrm{crystal}}$), and first ionization energy (${\mathrm{IE}}_{1}$), were considered to be the descriptor of HEAs for making ML. The $\delta$ and $\Delta \chi$ were made using Eqs. (2) and (3). Other features can be built as Eq. (4).

2 $$\updelta =100\sqrt{\sum_{{i}=1}^{{n}}{{{c}}_{{i}}(1-\frac{{{r}}_{{i}}}{\overline{{r}} })}^{2}},$$

3 $$\Delta\upchi =\sqrt{\sum_{{i}=1}^{{n}}{{{c}}_{{i}}({\upchi }_{{i}}-\overline{\upchi })}^{2},}$$

4 $$\mathrm{Feature}=\sum_{i=1}^{n}{c}_{i}({\mathrm{Feature})}_{i},$$

where ${r}_{i}$, ${\chi }_{i}$, and ${c}_{i}$ are the ${r}_{atomic}$, $\chi$, and concentration of element $i$. Weight-averaged features, $\overline{r }$ and $\overline{\chi }$ can be calculated using Eq. (4). The Pearson correlation coefficient of the features for ${\Delta E}_{f}$(FCC) is illustrated in Fig. 3. The results for ${\Delta H}_{f}$(BCC), *a*(FCC), and *a*(BCC) are shown in Figs. S1–S3. ${\Delta E}_{f}$(FCC) and ${\Delta E}_{f}$(BCC) demonstrated a weak relationship with the features. The *a*(FCC) and *a*(BCC) showed strong relation with VEC, ${V}_{mol}$, ${r}_{ionic}$, and ${r}_{\mathrm{VdW}}$. However, the overview of correlation between features and ${\Delta E}_{f}$ (and *a*) displayed both weak and strong values. Hence, the artificial neural network (ANN) was chosen for the supervised ML method because it can accomplish the complex and nonlinear features of the ML prediction model^12,67^. The 680 data were randomly split into 70% for the training set (476 data) and 30% for the test set (204 data points). The ANN consisted of four hidden layers, where each hidden layer contained thirteen. The Levenberg–Marquardt backpropagation^68^ was employed to train all models. The mean relative error (MRE) loop was used to improve the performance of prediction models, which can be calculated by Eq. (5).

5 $$\mathrm{MRE}=\frac{1}{n}\sum_{i=1}^{n}|\frac{{x}_{i}-{x}_{i,\mathrm{predicted}}}{{x}_{i}}|\times 100\%,$$

where the MRE cutoff is set to 5%. The prediction accuracy was also evaluated by the mean square error (MSE), mean absolute error (MAE), and coefficient of determination (${\mathrm{R}}^{2}$) as illustrated in Eqs. (6)–(8).

6 $$\mathrm{MSE}=\frac{1}{n}\sum_{i=1}^{n}{({x}_{i}-{x}_{i,\mathrm{predicted}})}^{2},$$

7 $$\mathrm{MAE}=\frac{1}{n}\sum_{i=1}^{n}\left|{x}_{i}-{x}_{i,\mathrm{predicted}}\right|,$$

8 $${\mathrm{R}}^{2}=\frac{\sum {({x}_{i,\mathrm{predicted}}-\overline{x })}^{2}}{\sum {\left({x}_{i,\mathrm{predicted}}-\overline{x }\right)}^{2}+\sum {\left({x}_{i}-{x}_{i,\mathrm{predicted}}\right)}^{2}}.$$

## Results and discussion

### DFT results

**Figure 4:**
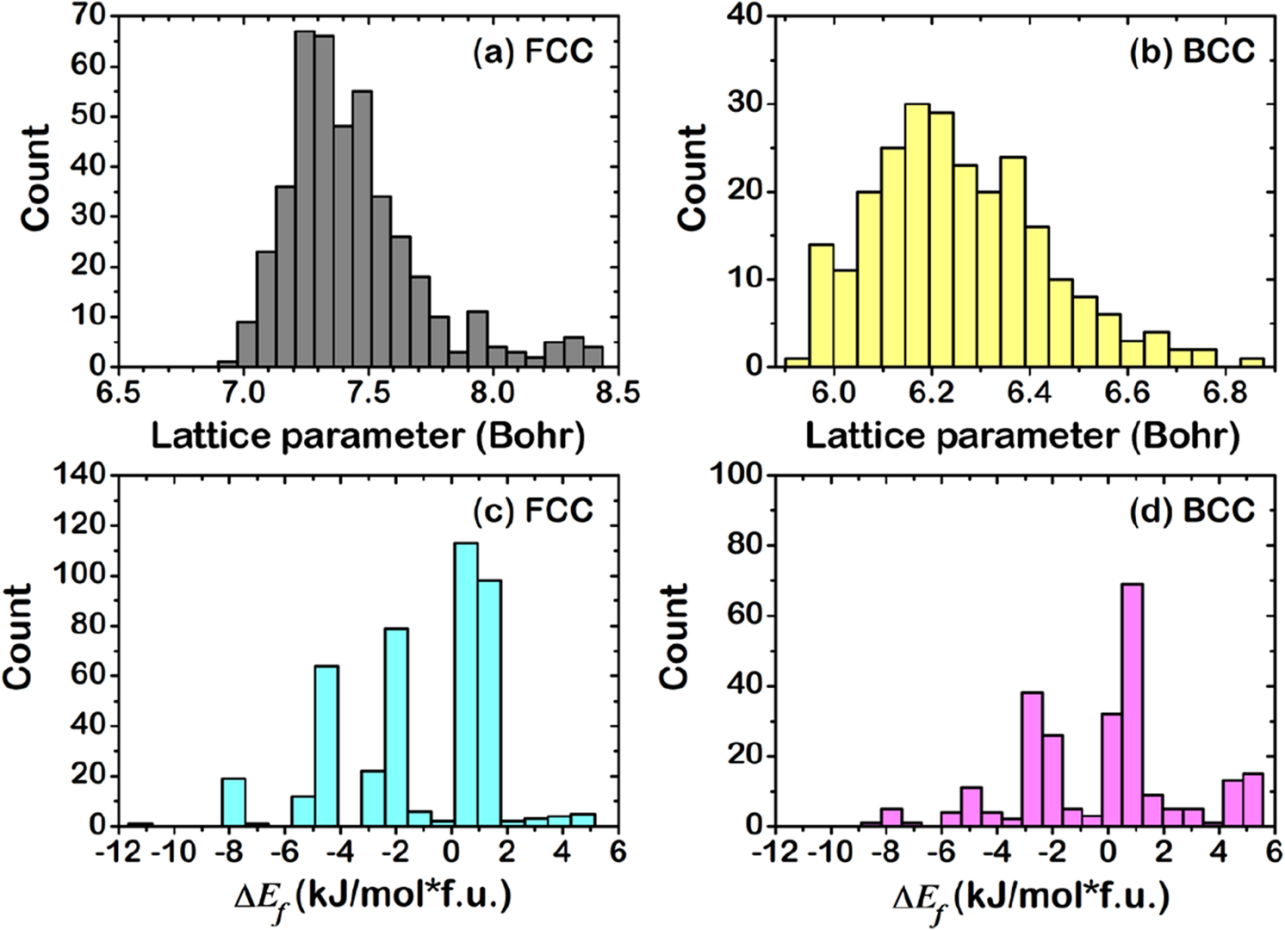
Distribution of the formation energy and lattice parameter given by DFT: (**a**) $a$(FCC), (**b**) $a$(BCC), (**c**) ${\Delta E}_{f}$(FCC), and (**d**) ${\Delta E}_{f}$(BCC).

**Figure 5:**
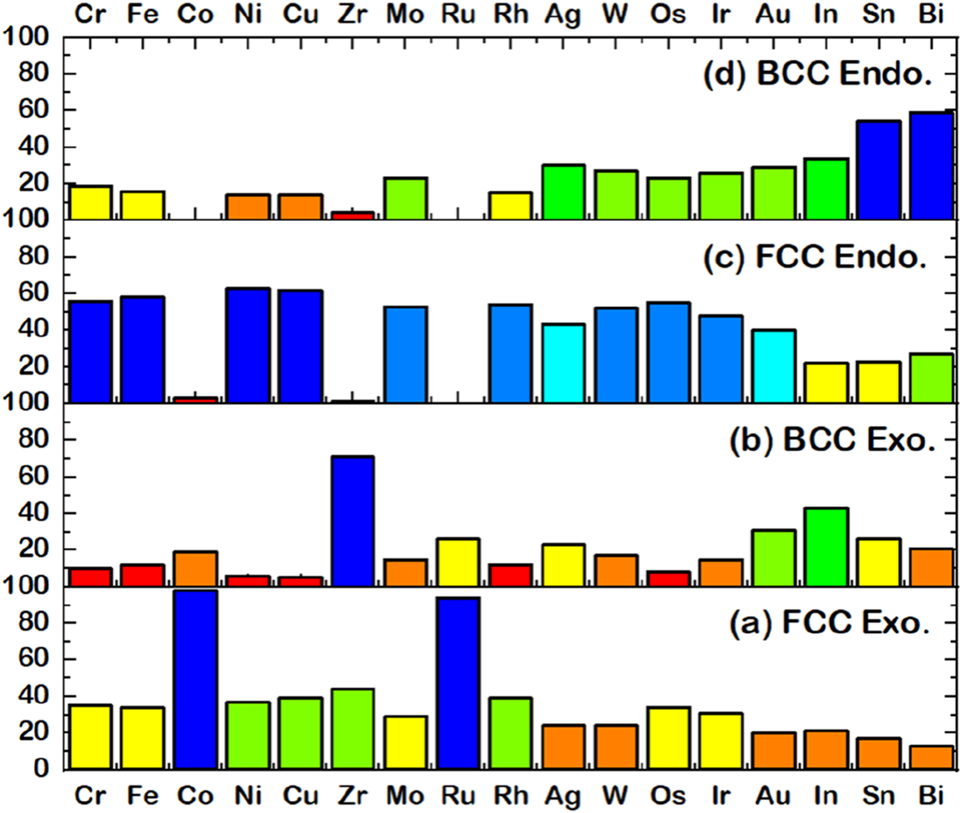
Distribution of elements counted from ${\Delta E}_{f}$ of PtPd*XYZ* HEAs (*X* $\ne$ *Y* $\ne$ *Z* = Cr, Fe, Co, Ni, Cu, Zr, Mo, Ru, Rh, Ag, W, Os, Ir, Au, In, Sn, Bi) through DFT-calculated results, including (**a**) FCC exothermic cases, (**b**) BCC exothermic cases, (**c**) FCC endothermic cases, and (**d**) BCC endothermic cases. The color classification in each group is based on the frequency of the system found to have either FCC Exo, BCC Exo, FCC Endo, or BCC Endo.

Figure S4 shows distribution data of DFT-calculated results of $a$(FCC), $a$(BCC), ${\Delta E}_{f}$(FCC), and ${\Delta E}_{f}$(BCC), while Fig. 4 chooses the more plausible phase. Each calculated HEA was classified into FCC or BCC phase by considering the ${\Delta E}_{f}$ to investigate the stable structure in thermodynamics^54,69^. For example, the DFT-calculated ${\Delta E}_{f}$ of PtPdCrFeCo HEA that is −2.6953 kJ/mol f.u. for FCC and −2.4593 kJ/mol f.u. for BCC indicates that FCC is thermodynamically favored over BCC due to the lowest ${\Delta E}_{f}$. Figure 4 shows the data distribution, retaining only the thermodynamically stable phase for each HEA. As a result, 680 HEAs include 431 FCC (~ 63%) and 249 BCC phase (~ 37%). Based on Hess’s law^70^, the positive and negative values of ${\Delta E}_{f}$ can be denoted to endothermic (Endo) and exothermic (Exo) reaction, respectively, composing 220 Endo (and 211 Exo) for FCC and 129 Endo (and 120 Exo) for BCC phases. This DFT-calculated data demonstrates the ratio of Endo:Exo close to 50%:50%, which is suitably balanced for creating ML prediction models. Both Endo and Exo HEAs were analyzed in terms of components. Figure 5 reveals the distribution of each element counted from Endo and Exo HEAs. Also, color-mapping was employed to separate the data group. The high concentration of each data is ordered as follows: blue, light blue, cyan, green, yellow, orange, and red. It was found that the Exo FCC HEAs have Co and Ru as the most frequent element (Fig. 5a). Similarly, Zr is a good candidate in Exo BCC HEAs (Fig. 5b). For Endo HEAs, Cr, Fe, Ni, Cu, Mo, Rh, Ag, W, Os, Ir, and Au hinder forming in the FCC phase, while Sn and Bi are the remarkable elements in the BCC phase.

It is noted that the KKR-CPA uses Green’s function method to calculate the electronic structure of alloys. In the KKR-CPA, the configuration average of the electronic structure is calculated^50–52^, hence, there is no detail of atomic configuration in the KKR-CPA method. Thus optimized lattice constant should be regarded as average lattice constant. Figure 4a, b reveal the histogram of *a*(FCC) and *a*(BCC). The lattice parameter of most FCC HEAs is distributed in 7.3–7.5 Bohr, while that of BCC HEAs shows a large distribution from 6.0 to 6.5 Bohr.

### ML-prediction models

**Figure 6:**
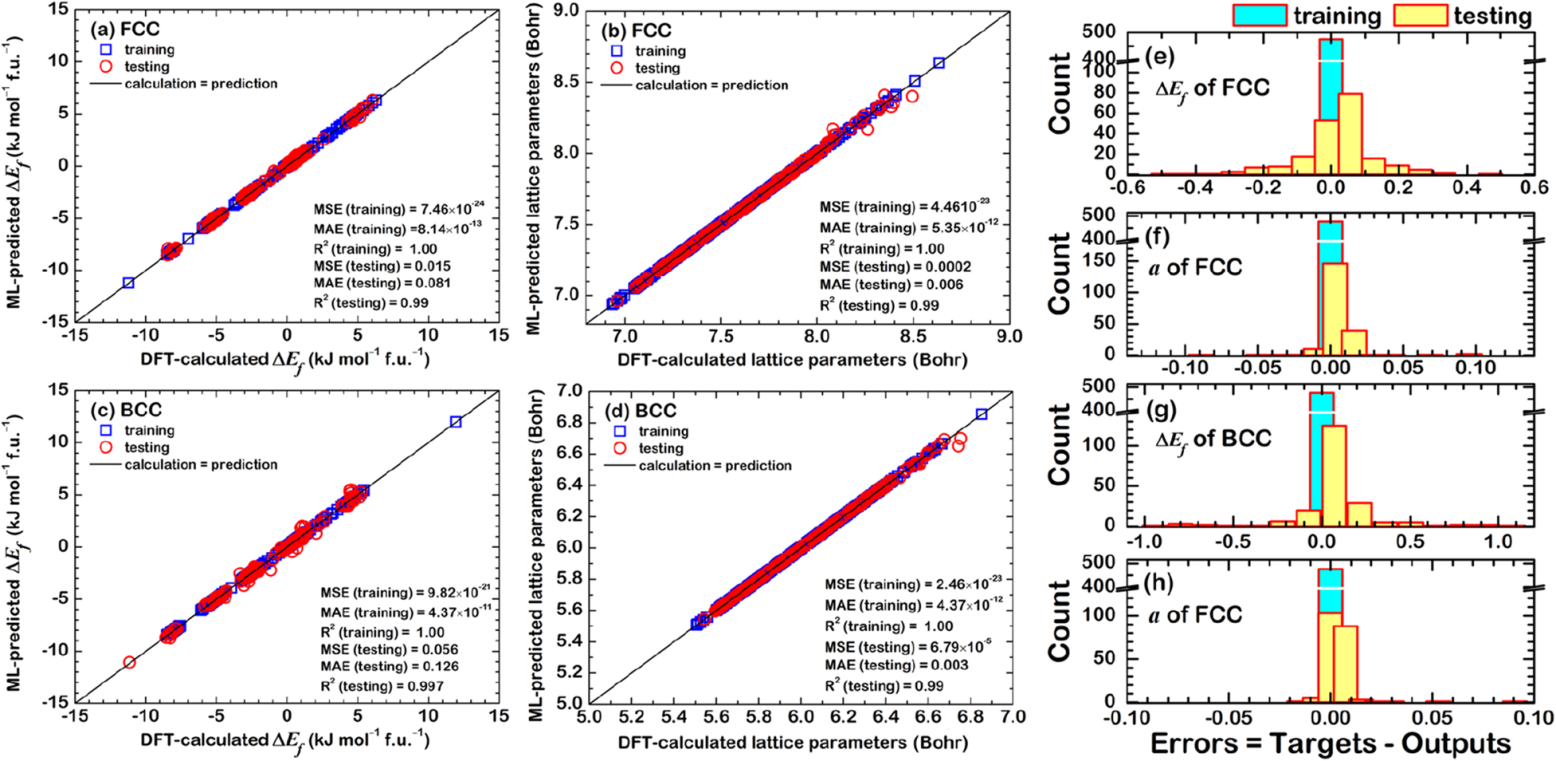
ML-predicted results compared with DFT-calculated data for (**a**) ${\Delta E}_{f}$(FCC), (**b**) ${\Delta E}_{f}$(BCC), (**c**) $a$(FCC), and (**d**) $a$(BCC). Error distribution in the testing is shown in (**e**)–(**h**).

The regression results are illustrated in Fig. 6, where DFT-calculated results were regressed through ANN. The MRE is 0.0237%, 0.0129%, 3.5554%, and 4.7429% for $a$(FCC), $a$(BCC), ${\Delta E}_{f}$(FCC), and ${\Delta E}_{f}$(BCC) prediction model, respectively. In the training results, the MSE and MAE values were close to zero and the ${\mathrm{R}}^{2}$ equal to 1 in all the cases, indicating that the ANN with the MRE-loop can improve the accuracy of the training. This also helped increase the accuracy of testing, which can be confirmed by 0.99 of ${\mathrm{R}}^{2}$. Figure 6e, g revealed the error values in the testing were ± 0.5 kJ/mol*f.u. for ${\Delta E}_{f}$(FCC) and ± 1.0 kJ/mol*f.u. for ${\Delta E}_{f}$(BCC). The error values of $a$ (Fig. 6f, h) were close to zero and seemed more accurate than the ${\Delta E}_{f}$ because of the strong correlation with features (see Figs. S2 and S3).

**Figure 7:**
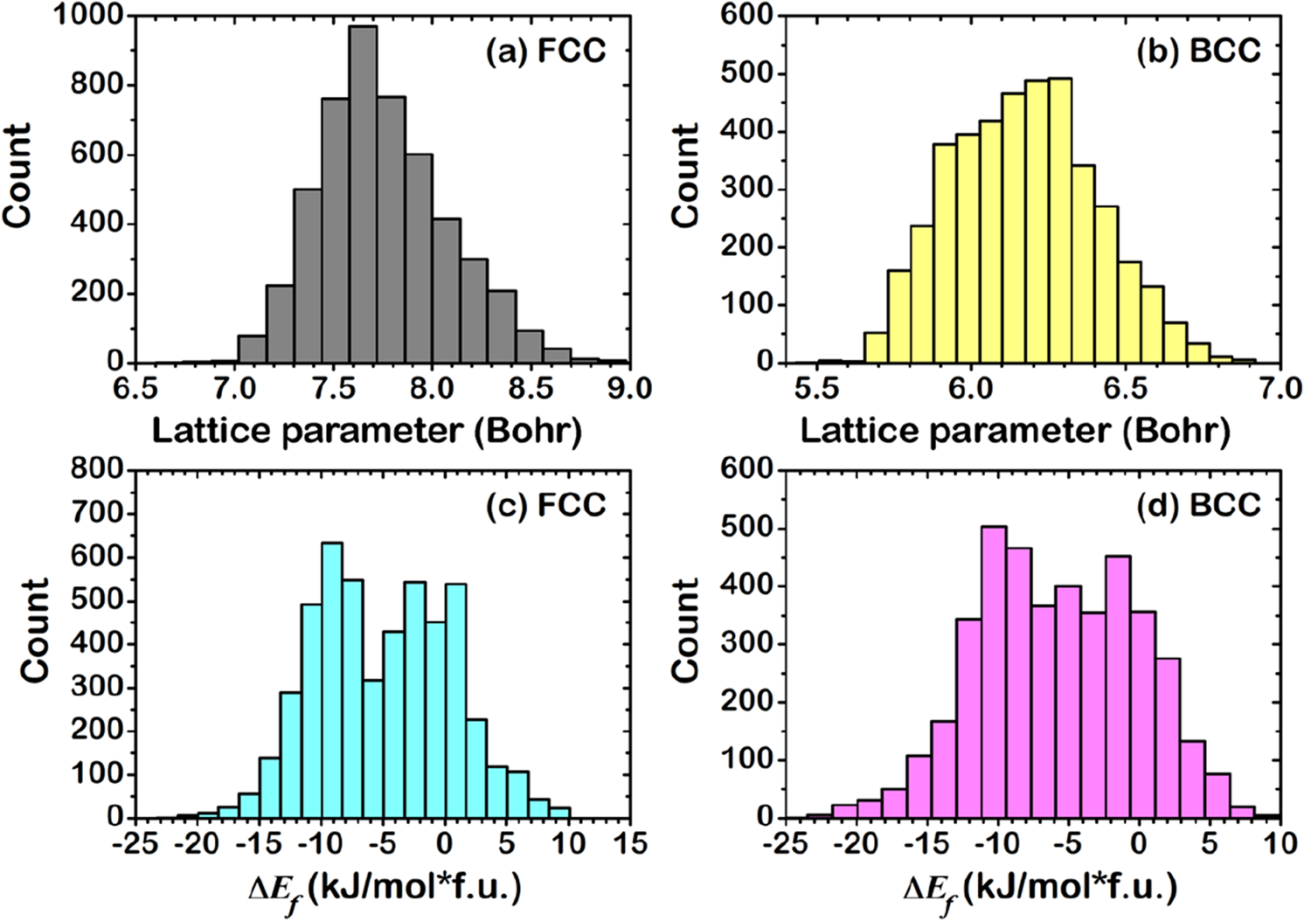
Distribution of ML-predicted results for (**a**) $a$(FCC), (**b**) $a$(BCC), (**c**) ${\Delta E}_{f}$(FCC), and (**d**) ${\Delta E}_{f}$(BCC).

The built ML models are employed to predict the 9,139 HEAs as the formulae of Pt_0.2_Pd_0.2_*X*_0.2_*Y*_0.2_*Z*_0.2_ (*X* $\ne$ *Y* $\ne$ *Z*), where *X*, *Y*, and *Z* are considered from 39 elements: Sc, Ti, V, Cr, Mn, Fe, Co, Ni, Cu, Zn, Y, Zr, Nb, Mo, Tc, Ru, Rh, Ag, Cd, Hf, Ta, W, Re, Os, Ir, Au, Hg, Al, Si, P, Ga, Ge, As, In, Sn, Sb, Tl, Pb, and Bi. The ML-predicted results of $a$(FCC), $a$(BCC), ${\Delta E}_{f}$(FCC), and ${\Delta E}_{f}$(BCC) without distinguishing the phase are shown in Fig. S5. The thermodynamically stable phase of 9,139 HEAs is illustrated in Fig. 7. Figure 7a,b reveal enlarged distribution: 7.4–8.0 Bohr for $a$(FCC) and 5.9–6.5 Bohr for $a$(BCC) due to the variety of atomic radius of elements. Similarly, ${\Delta E}_{f}$ is distributed between about −25.0 kJ/mol f.u. and + 10.0 kJ/mol f.u. for both FCC and BCC. The number of FCC and BCC HEAs is 5002 and 4137, respectively. The number of Exo HEAs is 4140 for FCC and 3567 for BCC phase, more than the Endo HEAs, 862 for FCC and 570 for BCC phase.

**Figure 8:**
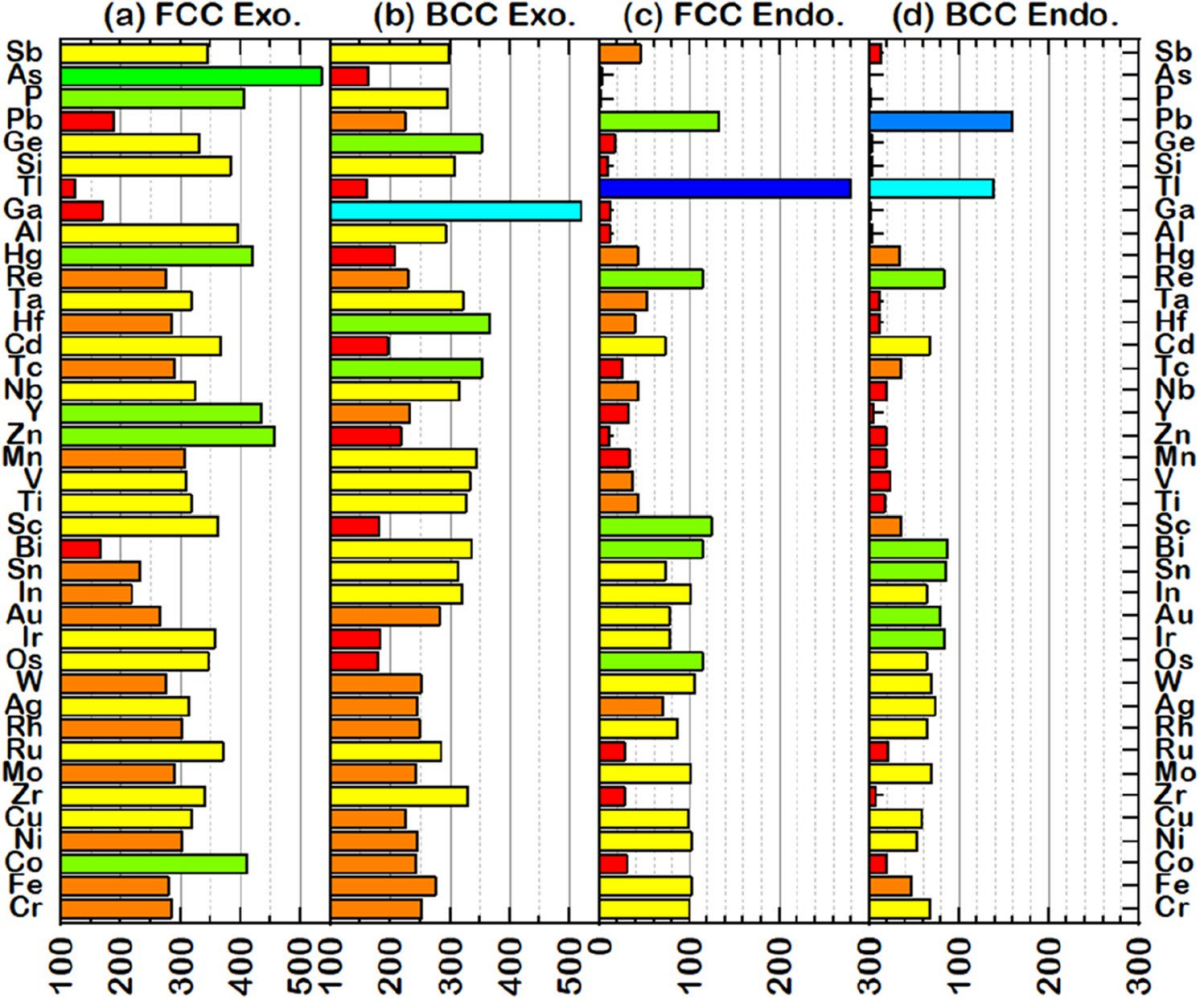
Distribution of elements counted from ${\Delta E}_{f}$ of PtPd*XYZ* HEAs (*X* $\ne$ *Y* $\ne$ *Z* = Sc, Ti, V, Cr, Mn, Fe, Co, Ni, Cu, Zn, Y, Zr, Nb, Mo, Tc, Ru, Rh, Ag, Cd, Hf, Ta, W, Re, Os, Ir, Au, Hg, Al, Si, P, Ga, Ge, As, In, Sn, Sb, Tl, Pb, Bi) through ML-predicted results: (**a**) FCC exothermic cases, (**b**) BCC exothermic cases, (**c**) FCC endothermic cases, and (**d**) BCC endothermic cases. The color classification in each group is based on the frequency of the system found to have either FCC Exo, BCC Exo, FCC Endo, or BCC Endo.

**Figure 9:**
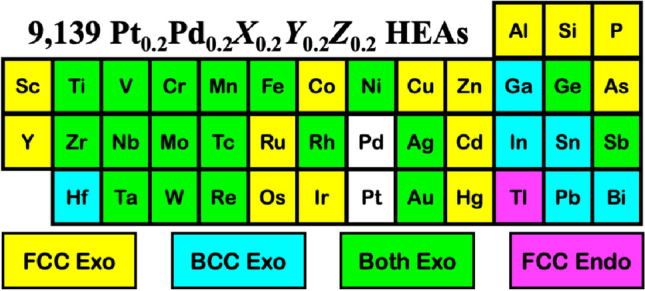
Predicted role of individual elements in the HEA formation energy and phase.

To understand the role of each element involving Endo and Exo forms, Fig. 8 illustrates the element distribution obtained from 9139 PtPd*XYZ* HEAs (*X* $\ne$ *Y* $\ne$ *Z* = Sc, Ti, V, Cr, Mn, Fe, Co, Ni, Cu, Zn, Y, Zr, Nb, Mo, Tc, Ru, Rh, Ag, Cd, Hf, Ta, W, Re, Os, Ir, Au, Hg, Al, Si, P, Ga, Ge, As, In, Sn, Sb, Tl, Pb, Bi). Overall, all elements of choice except Tl and Pb are found to form Endo HEAs as the ratio FCC:BCC of 50%:50%. Co, Zr, Zn, Al, Ga, Si, Ge, P, and As are hardly involved in Endo HEAs, contrary to Tl, which favors forming the FCC Endo HEAs (Fig. 8c). For the Exo HEAs, the green bar in the FCC phase and the cyan bar in the BCC phase indicate high distribution. In Exo FCC HEAs (Fig. 8a), Co, Zn, Y, Hg, P, and As can be totaled more than 400 HEAs, followed by Sc, Ti, V, Cu, Zr, Nb, Ru, Ag, Cd, Ta, Os, Ir, Si, Ge, and Sb counted by more than 300 HEAs (yellow bar). For the Exo BCC HEAs shown in Fig. 8b, the elements found in more than 300 HEAs are Ti, V, Mn, Y, Zr, Nb, Tc, Hf, Ta, Si, Ga, Ge, In, Sn, and Bi. Among these, Ga, the only element that naturally forms as an orthorhombic structure, plays a significant role in the BCC phase found in more than 500 Exo HEAs (the cyan bar in Fig. 8b). The phase of HEAs is summarized in Fig. 9. Among these data, 15 and 6 elements favor forming FCC and BCC, respectively, while the others almost count both FCC and BCC phases.

The literature on PtPd-based HEAs is hardly found because they are new group materials. Based on the available data, the phase classification using ${\Delta E}_{f}$ is demonstrated in Table 1. PtPd-based HEAs of five principal elements reported in the literature showed the predominance of the FCC phase. The ${\Delta E}_{f}$ values given by ANN models predicted the thermodynamic propensity of the FCC formation of these PtPd-based HEAs, thus agreeing with the available experimental data. In addition to FCC HEAs, BCC HEAs experimentally reported in literature such as CrFeCoNiAl^56,58^, MnFeNiSiGa^56^, NbMoTaW^54,62,63^, VNbMoTaW^54,62,63^, TiVCrFeCoNiCuAl^64^, TiZrNbHfTa^61,65^, and TiZrNbMo^58^ were employed to validate the KKR-CPA method (Tables S1 and S2). In this case, calculated ${\Delta E}_{f}$ values successfully categorized the correct phase.

**Table 1:** Phase classification of PtPd-based HEAs by ${\Delta E}_{f}$ (in kJ mol^–1^ f.u.^–1^) with *a* (in Å) validated with literature data.

HEAs	${\Delta E}_{f}$ (FCC)	*a *(FCC)	${\Delta E}_{f}$ (BCC)	*a *(BCC)	Lowest ${\Delta E}_{f}$	Predicted phase	References
PtPdFeCoNi	−2.6973	3.6713	−2.4330	2.9140	−2.6973	FCC	FCC^26,36^ *a* = 3.73^36^
PtPdFeCoIr	−2.6859	3.7440	−2.376	2.9715	−2.6859	FCC	FCC^37^
PtPdFeRhIr	0.2036	3.8130	0.4338	3.0264	0.2036	FCC	FCC^38^ *a* = 3.834^38^
PtPdCoNiCu	−2.6009	3.6831	−2.3553	2.9233	−2.6009	FCC	FCC^39^
PtPdCuAgAu	0.0882	3.9351	0.1317	3.1233	0.0882	FCC	FCC^7,8,33^ *a* = 3.936^7^
PtPdRuRhIr	−5.2674	3.8669	−4.9991	3.0692	−5.2674	FCC	FCC^34,35,42,43^ *a* = 3.8560^35^ *a* = 3.82^36^ *a* = 3.84 – 3.96^42^
PtPdRuAgIr	−5.0594	3.9276	−4.9444	3.1173	−5.0594	FCC	FCC^34,41^
PtPdRuRhAu	−5.2834	3.9165	−5.1907	3.1085	−5.2834	FCC	FCC^40^

**Figure 10:**
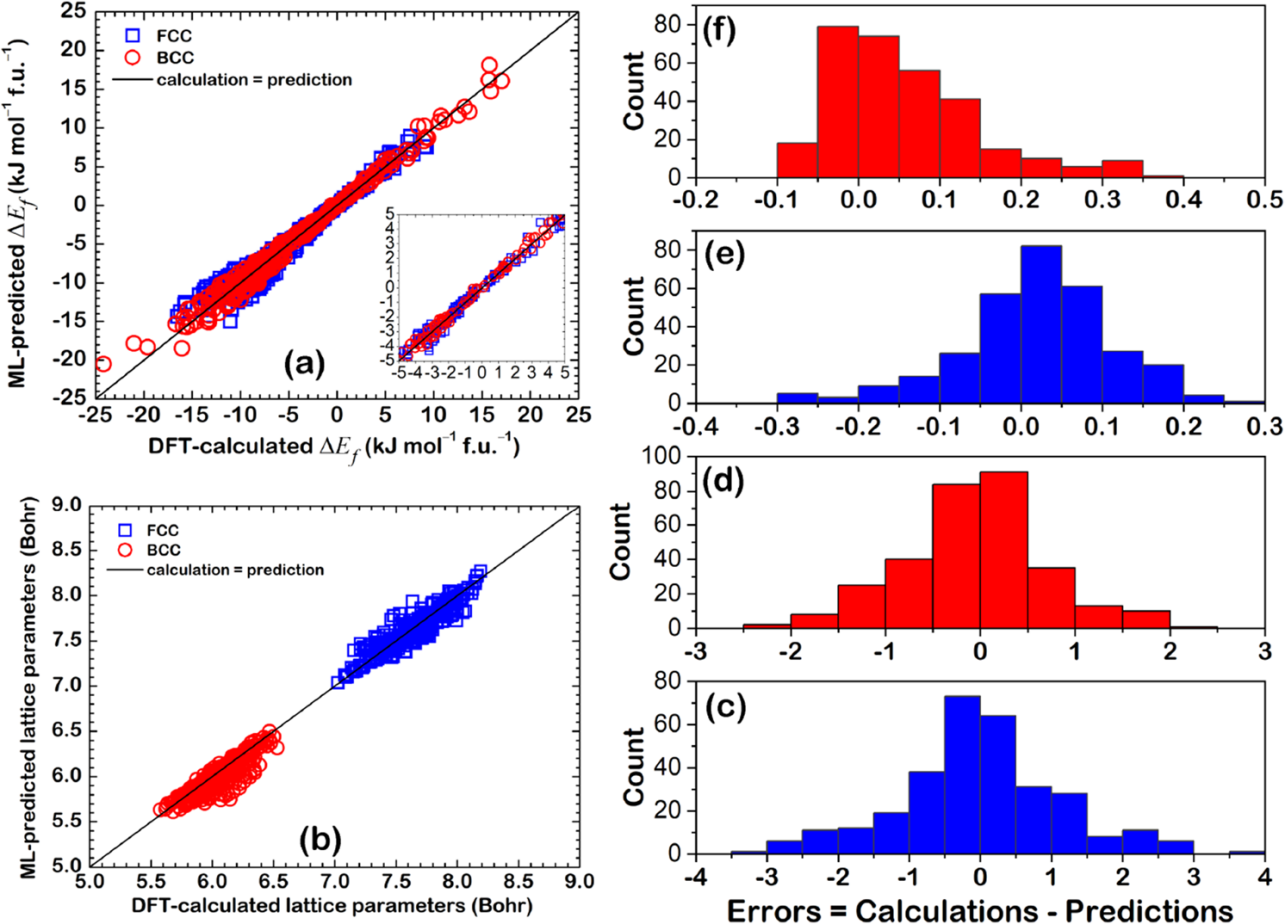
Validation of PtPdCr*XY*, PtPdNiSn*X*, and PtPd*XYZ* HEAs including regression plots of (**a**) formation energy, (**b**) lattice parameters, (**c**) error of formation energy in FCC data, (**d**) error of formation energy in BCC data, (**e**) error of lattice parameters in FCC data, (**f**) error of lattice parameters in BCC data.

The prediction on the HEAs containing untrained elements such as Sc, Ti, V, Mn, Zn, Y, Nb, Tc, Cd, Hf, Ta, Re, Hg, Al, Si, P, Ga, Ge, As, Sb, Te, and Pb was tested by the calculated ${\Delta E}_{f}$ and *a* of PtPdCr*XY*, PtPdNiSn*X*, and PtPd*XYZ*. This aims to examine whether these prediction models are accurate. Because the DFT calculation of 9,139 HEAs includes 36,556 data for ${\Delta E}_{f}$(FCC), ${\Delta E}_{f}$(BCC), *a*(FCC), and *a*(BCC), the selected HEAs composing untrain elements are used to reduce computational time. The formulae PtPdNiSn*X*, PtPdCr*XY,* and PtPd*XYZ* are represented the HEAs containing one, two, and three untrained elements. The elements *X* and *Y* in PtPdNiSn*X* and PtPdCr*XY* are Sc, Ti, V, Mn, Zn, Y, Nb, Tc, Cd, Hf, Ta, Re, Hg, Al, Si, P, Ga, Ge, As, Sb, Te, Pb. The elements *X*, *Y*, and *Z* in PtPd*XYZ* are Sc, Ti, V, Mn,Zn, Al, Si, Sb. The 309 HEAs for validation are implemented in Table S3. The regression plot between DFT-calculation and ML-prediction is illustrated in Fig. 10a,b. The MAE, MSE, and R^2^ are listed in Table 2. Although the MSE and MAE of the untrained data (Table 2) are higher than that of the trained data (Fig. 6a,c), the R^2^ of all ${\Delta E}_{f}$ from the untrained data is still as high as 0.99. The predicted ${\Delta E}_{f}$ of the selected HEAs has an error ± 4 and ± 3 kJ mol^–1^ f.u.^–1^ for FCC and BCC prediction model, respectively (Fig. 10c,d). Figure 10b shows the regression plot of *a* that seems less accuracy. Figure 10e,f indicate the error value of predicted *a* less than ± 0.4 Å. This error in *a* is in an acceptable range for the bulk structure, although the R^2^ of untrained *a* is less than that of the trained *a* (Fig. 6b,d). In addition to the R^2^, the MSE and MAE values as well as the error value in the prediction should be considered when evaluating the performance of prediction. As a result, the MSE and MAE of untrained *a* are in the same range of that the trained *a* (Fig. 6b,d). Although there are some errors found on the prediction of selected HEAs, the performance of prediction models is accepted.

**Table 2:** MSE, MAE, and R^2^ of formation energy (${\Delta E}_{f}$) and lattice parameters (*a*) for the predicted PtPdCr*XY*, PtPdNiSn*X*, and PtPd*XYZ.*

	${\Delta E}_{f}$ (FCC)	*a *(FCC)	${\Delta E}_{f}$ (BCC)	*a *(BCC)
MSE	1.31	0.009(2)	0.58	0.011(5)
MAE	1.31	0.005(4)	0.58	0.000(2)
R^2^	0.98(1)	0.86(7)	0.98(7)	0.75(9)

## Conclusion

A combination of DFT and ANN was employed to predict the possible formulae of penta-metallic high-entropy alloys. The formation energy and lattice parameter of each system were determined via the KKR-CPA method. The ANN was employed to construct the prediction models and speed up the material screening. The training-to-testing data are 70%:30%, derived from DFT-calculated data of 680 HEAs from 17 elements. 15 features were used in such an algorithm are VEC, $M$, ${r}_{atomic}$, $\delta$, $\chi$, $\Delta \chi$, ${E}_{ea}$, $\rho$, ${V}_{mol}$, ${T}_{mel}$, ${\Delta H}_{f,\mathrm{atom}}$, ${r}_{ionic}$, ${r}_{\mathrm{VdW}}$, ${r}_{\mathrm{crystal}}$, and ${\mathrm{IE}}_{1}$. The built models possessed high accuracy in the testing, accompanied with the ${\mathrm{R}}^{2}$ values close to unity and MRE within 5%. Based on the prediction models, 9,139 PtPd-based HEA systems created from a pool of 39 elements were classified into 5,002 FCC and 4,137 BCC systems; the HEA screening rules can be summarized as follows.

i. HEAs with the component of Sc, Co, Cu, Zn, Y, Ru, Cd, Os, Ir, Hg, Al, Si, P, As, and Tl have a high tendency to form in the FCC phase.
ii. HEAs with the component Hf, Ga, In, Sn, Pb, and Bi have a high tendency to form in the BCC phase.
iii. Ti, V, Cr, Mn, Fe, Ni, Zr, Nb, Mo, Tc, Rh, Ag, Ta, W, Re, Au, Ge, and Sb have a comparable tendency to form in either FCC or BCC phase.

These screening rules applied in this work provide the fundamental for the discovery of bulk HEA, where the development of algorithms for the screening of HEA in terms of stable surface configuration is the outlook for future work.

## Supplementary Information

Supplementary Information.

## Data Availability

The authors declare that relevant data are within the manuscript.
