# Supplementary material for: High-throughput materials screening algorithm based on first-principles density functional theory and artificial neural network for high-entropy alloys
Source: Sci Rep. 2022 Oct 5;12:16653. doi: 10.1038/s41598-022-21209-0 (PMC9534987; doi:10.1038/s41598-022-21209-0)
Supplement: Supplementary file 1 — Supplementary Information. [file 41598_2022_21209_MOESM1_ESM.pdf]

Supporting Information

High-throughput materials screening algorithm based on first-principles density functional theory and artificial neuron network for high-entropy alloys

Meena Rittiruam[1,2,3], Jakapob Noppakhun[1,2,3], Sorawee Setasuban[1,2,4], Nuttanon Aumnongpho[1,2,3], Attachai Sriwattana[1,2,4], Suphawich Boonchuay[1,2,4], Tinnakorn Saelee[1,2,4], Chanthip Wangphon[1,2,4], Annop Ektarawong[5,6], Patchanee Chammingkwan[7], Toshiaki Taniike[7], Supareak Praserthdam[1, 2, *], Piyasan Praserthdam[2]

[1]*High-Performance Computing Unit (CECC-HCU), Center of Excellence on Catalysis and Catalytic Reaction Engineering (CECC), Chulalongkorn University, Bangkok 10330, Thailand*

[2]*Center of Excellence on Catalysis and Catalytic Reaction Engineering (CECC), Chulalongkorn University, Bangkok 10330, Thailand*

[3]*Rittiruam Research Group, Chulalongkorn University, Bangkok 10330, Thailand*

[4]*Saelee Research Group, Chulalongkorn University, Bangkok 10330, Thailand*

[5]*Extreme Conditions Physics Research Laboratory and Center of Excellence in Physics of Energy Materials (CE:PEM), Department of Physics, Faculty of Science, Chulalongkorn University, Bangkok, 10330, Thailand*

[6]*Chula Intelligent and Complex Systems, Faculty of Science, Chulalongkorn University, Bangkok, 10330, Thailand*

[7]*Graduate School of Advanced Science and Technology, Japan Advanced Institute of Science and Technology, 1-1 Asahidai, Nomi, Ishikawa 923-1292, Japan*

Corresponding author email: supareak.p@chula.ac.th (Supareak Praserthdam)

Table of Contents

1. Validation of KKR-CPA method for multi-component alloys 3

2. Supporting results 6

References 21

List of Supporting Tables

Table S1 Enthalpy of formation energy (ΔHf) in eV/atom and lattice parameters in Å of NbMoTaWV calculated by DFT+SQS and KKR–CPA method. 4

Table S2 Enthalpy of formation energy (ΔHf) in eV/atom of various alloys calculated by KKR–CPA method. 5

Table S3 Compared lattice parameters and enthalpy of formation energy (ΔHf). These HEAs are PtPdCrXY (X and Y = Sc, Ti, V, Mn, Zn, Y, Nb, Tc, Cd, Hf, Ta, Re, Hg, Al, Sil, P, Ga, Ge, As, Sb, Te, Pb), PtPdNiSnX (X = Sc, Ti, V, Mn, Zn, Y, Nb, Tc, Cd, Hf, Ta, Re, Hg, Al, Sil, P, Ga, Ge, As, Sb, Te, Pb), and PtPdXYZ (X, Y, and Z = Sc, Ti, V, Mn,Zn, Al, Si, Sb)... 9

List of Supporting Figures

Figure S1 Heat-map Pearson correlation coefficient of features for ΔHf(BCC) of HEAs. 6

Figure S2 Heat-map Pearson correlation coefficient of features for a(FCC) of PtPd-based HEAs. 6

Figure S3 Heat-map Pearson correlation coefficient of features for a(BCC) of PtPd-based HEAs. 7

Figure S4 Distribution of DFT-calculated results for (a) a(FCC), (b) a(BCC), (c) ΔHf(FCC), and (d) ΔHf(BCC) for individual compositions. 7

Figure S5 Distribution of ML-predicted results for (a) a(FCC), (b) a(BCC), (c) ΔHf(FCC), and (d) ΔHf(BCC) for individual compositions 8

1. Validation of KKR-CPA method for multi-component alloys

Troparevsky *et al*. [1] demonstrated that the KKR-CPA method provided accurate results for multi-component alloys. The fcc CrFeCoNi supercell with 2,048 atoms was calculated by the locally self-consistent multiple scattering (LSMS) method. The calculated magnetic moments of the KKR-CPA are closed to that of the LSMS method. Hu *et al*. experimentally prepared NbMoTaWV high-entropy alloy (HEA) using vacuum arc melting, which obtained BCC structure from the X-ray diffraction result [2]. The formation energy and lattice parameters were calculated using the density functional theory to validate the HEA containing five elements. The authors [2] designed the HEA model using a special quasi-random supercell (SQS) method. Our calculation is based on the KKR-CPA method through the Akai-KKR (Machikaneyama) package [3, 4], where the computational details are revealed in section 2 of this paper. As shown in **Table S1**, the KKR-CPA method provided the calculation results close to the experimental data [2]. In addition, the enthalpy of formation energy (ΔH_f) via KKR-CPA is the same trend that results via the SQS method. The BCC structure showed the lowest ΔH_f which is the thermodynamically stable phase of the NbMoTaWV HEA. Other single-phase alloys found in literature data were used to validate the accuracy of KKR-CPA implemented in the Akai-KKR package, as shown in **Table S2**. As a result, the KKR-CPA can indicate the FCC and BCC phase stability of multi-component alloys.

Table S1 Enthalpy of formation energy (ΔH_f) in eV/atom and lattice parameters in Å of NbMoTaWV calculated by DFT+SQS and KKR–CPA method.

NbMoTaWV	Formation energy	Lattice parameters
BCC	-0.092 (DFT+SQS)*<br>0.423 (KKR-CPA)**	a = 3.185 (experiment)*<br>a = 3.167 (DFT+SQS)*<br>a = 3.203 (KKR-CPA)**
FCC	0.406 (DFT+SQS)*<br>1.107 (KKR-CPA)**	-<br>a = 4.089 (KKR-CPA)**
HCP	0.563 (DFT+SQS)*<br>2.647 (KKR-CPA)**	-<br>a = 2.780, c/a = 1.1 (KKR-CPA)**
Experimental data (BCC)	-	a = 3.185*

* Reference [2].

** This work.

Table S2 Enthalpy of formation energy (ΔH_f) in eV/atom of various alloys calculated by KKR–CPA method.

Alloys	ΔH_f(FCC)	ΔH_f(BCC)	Lowest ΔH_f	Phase	Reference
CrFeCoNi	-0.888	-0.830	-0.888	FCC	FCC [5, 6]
CrFeCoNiAl	-5.768	-5.794	-5.794	BCC	BCC [5, 7]
CrFeCoNiCu	-0.042	0.351	-0.042	FCC	FCC [5]
CrFeCoNiPd	-1.923	-1.847	-1.923	FCC	FCC [8]
CrMnCoNi	-3.028	-2.988	-3.028	FCC	FCC [9]
CrMnFeCoNi	-0.698	-0.653	-0.698	FCC	FCC [5, 9, 10]
CrMnFeCoNiCu	-0.050	0.287	-0.050	FCC	FCC [10]
CrMnFeCoNiNb	-0.704	-0.431	-0.704	FCC	FCC [10]
CrMnFeCoNiV	-0.949	-0.660	-0.949	FCC	FCC [10]
FeCoNi	-1.793	-1.291	-1.793	FCC	FCC [5]
MnFeCoNi	-1.297	-1.030	-1.297	FCC	FCC [5, 9]
MnFeNiSiGa	-1.906	-2.149	-2.149	BCC	BCC [5]
NbMoTaW	0.951	0.302	0.302	BCC	BCC [2, 11, 12]
TiCrFeCoNi	-2.814	-2.699	-2.814	FCC	FCC [5]
TiVCrFeCoNiCuAl	-1.953	-1.978	-1.978	BCC	BCC [13]
TiZrNbHfTa	-5.434	-5.671	-5.671	BCC	BCC [10, 14]
TiZrNbMo	-4.066	-4.380	-4.380	BCC	BCC [7]
VNbMoTaW	1.107	0.423	0.423	BCC	BCC [2, 11, 12]

2. Supporting results

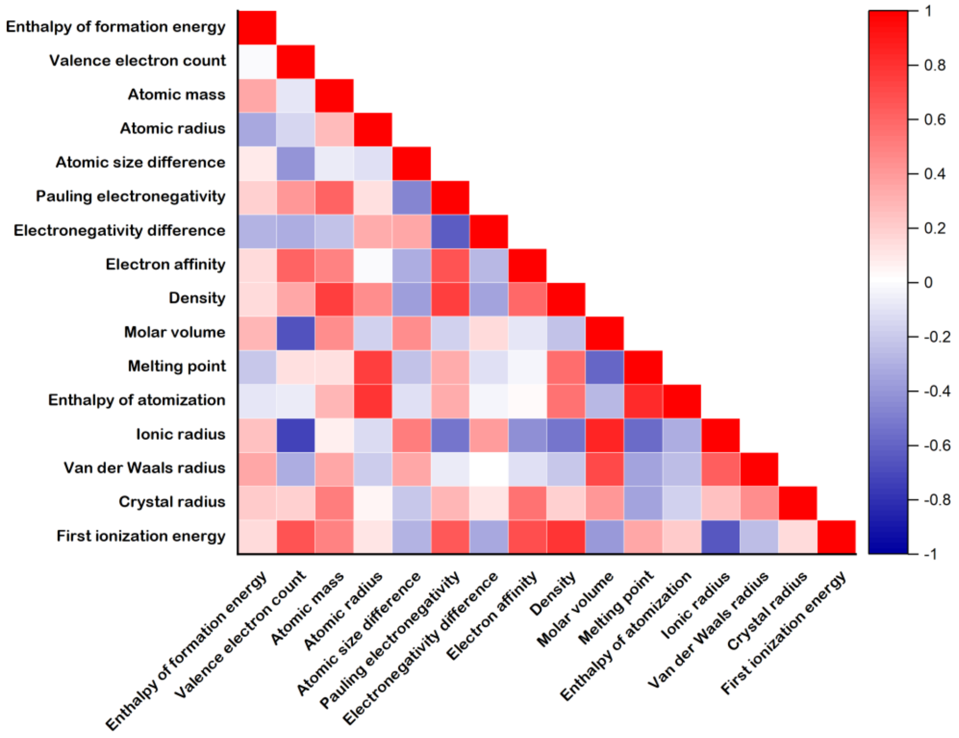

Figure S1 Heat-map Pearson correlation coefficient of features for ΔH_f(BCC) of HEAs.

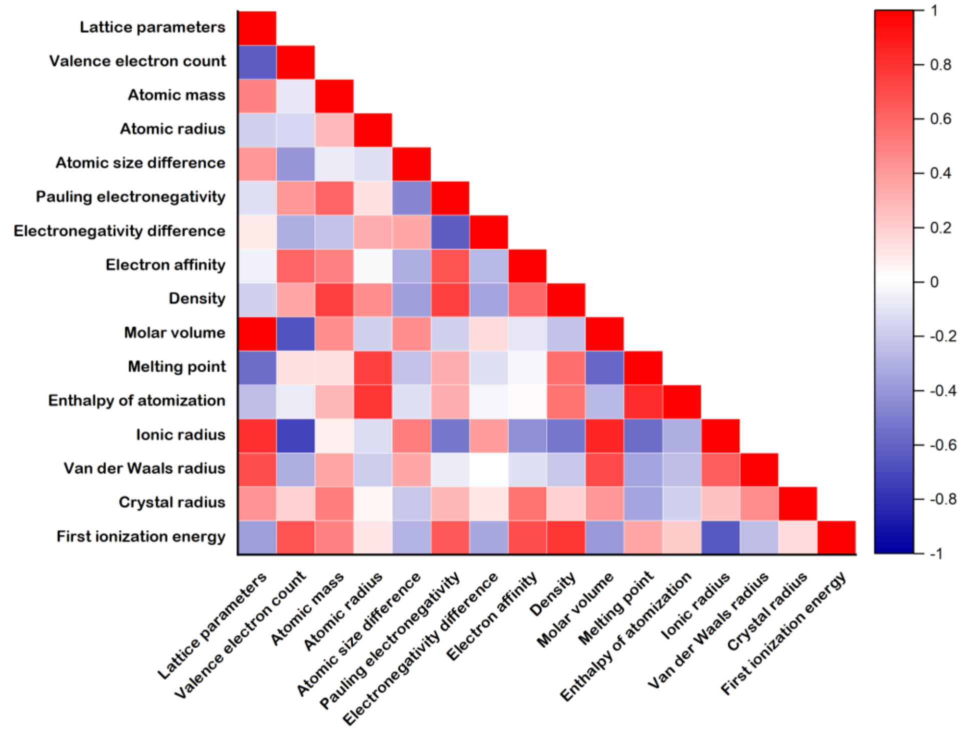

Figure S2 Heat-map Pearson correlation coefficient of features for a(FCC) of PtPd-based HEAs.

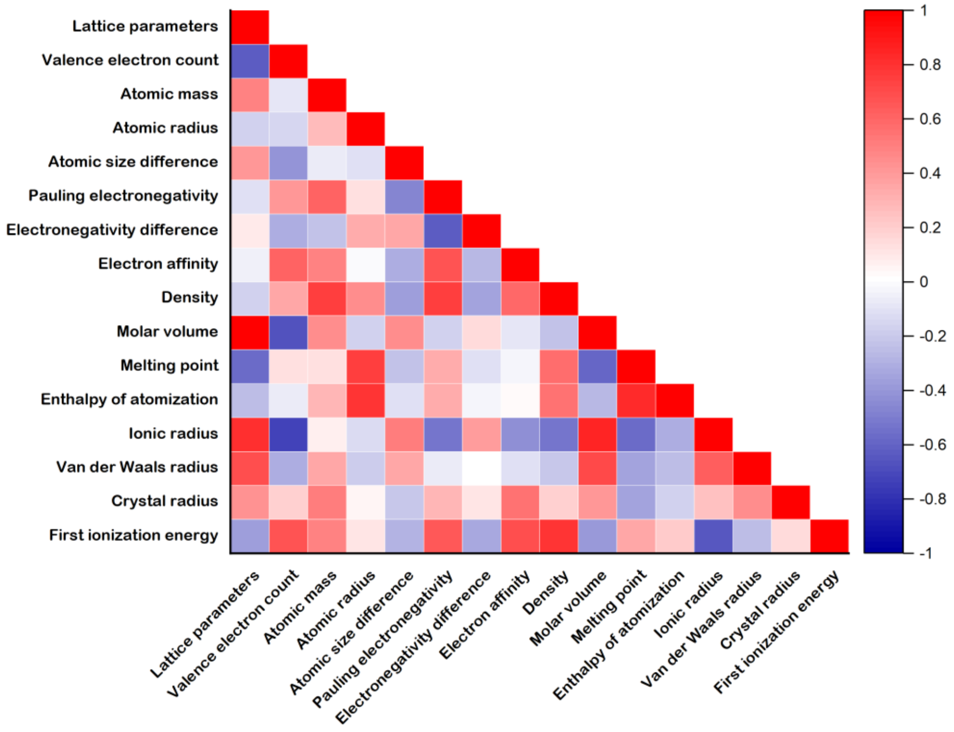

Figure S3 Heat-map Pearson correlation coefficient of features for a(BCC) of PtPd-based HEAs.

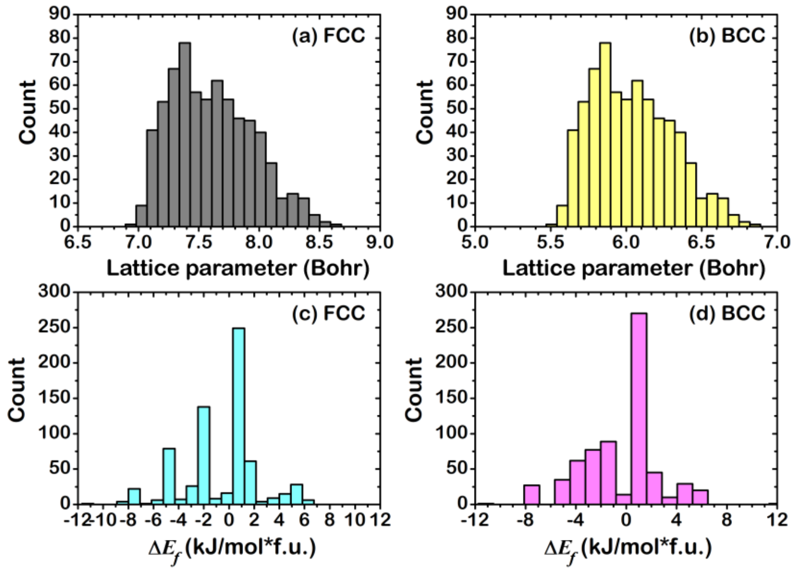

Figure S4 Distribution of DFT-calculated results for (a) a(FCC), (b) a(BCC), (c) ΔH_f(FCC), and (d) ΔH_f(BCC) for individual compositions.

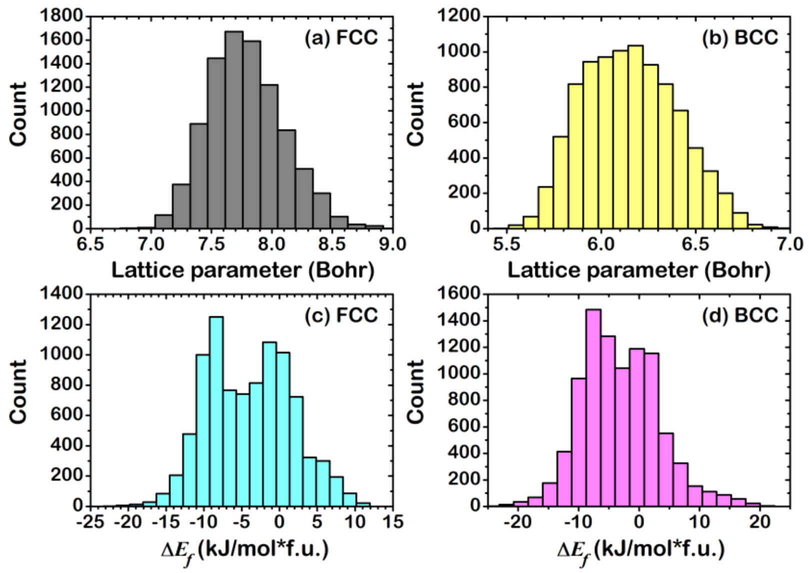

Figure S5 Distribution of ML-predicted results for (a) a(FCC), (b) a(BCC), (c) ΔH_f(FCC), and (d) ΔH_f(BCC) for individual compositions.

Table S3 Compared lattice parameters and enthalpy of formation energy (ΔH_f). These HEAs are PtPdCrXY (X and Y = Sc, Ti, V, Mn, Zn, Y, Nb, Tc, Cd, Hf, Ta, Re, Hg, Al, Sil, P, Ga, Ge, As, Sb, Te, Pb), PtPdNiSnX (X = Sc, Ti, V, Mn, Zn, Y, Nb, Tc, Cd, Hf, Ta, Re, Hg, Al, Sil, P, Ga, Ge, As, Sb, Te, Pb), and PtPdXYZ (X, Y, and Z = Sc, Ti, V, Mn,Zn, Al, Si, Sb).

HEAs	Lattice parameters (Bohr)						ΔH_f (kJ/mol*f.u.)						Phase	
	FCC Cal.	FCC ML	BCC Cal.	BCC ML	FCC error	BCC error	FCC Cal.	FCC ML	BCC Cal.	BCC ML	FCC error	BCC error	Cal.	ML
PtPdCrScTi	7.557	7.5023	5.998	5.9761	0.0547	0.0219	0.37731	0.3628	2.8135	3.0582	0.01451	-0.24466	FCC	FCC
PtPdCrScV	7.448	7.3888	5.9115	5.8934	0.05922	0.01809	0.4526	0.5453	3.271	3.5554	-0.0927	-0.28443	FCC	FCC
PtPdCrScMn	7.3752	7.3472	5.8537	5.8518	0.02803	0.00193	0.92072	0.8939	3.8791	4.5106	0.02682	-0.63148	FCC	FCC
PtPdCrScZn	7.497	7.4233	5.9504	5.9401	0.07368	0.01026	-5.427	-4.523	0.7984	0.7604	-0.9045	0.03802	FCC	FCC
PtPdCrScY	7.9496	8.0232	6.3096	6.294	-0.07358	0.01562	-3.5014	-3.61	11.202	10.277	0.10829	0.924966	FCC	FCC
PtPdCrScNb	7.6085	7.5367	6.0388	5.9898	0.07176	0.04904	-6.8036	-6.542	1.227	1.3943	-0.2617	-0.16732	FCC	FCC
PtPdCrScTc	7.5026	7.4479	5.9548	5.9299	0.05471	0.02493	0.74088	0.9261	3.1039	3.1999	-0.1852	-0.096	FCC	FCC
PtPdCrScCd	7.7399	7.6051	6.1431	6.1333	0.13477	0.00984	3.11563	2.4925	7.5849	8.3351	0.62313	-0.75016	FCC	FCC
PtPdCrScHf	7.7513	7.6937	6.1522	6.1162	0.05763	0.03603	1.76035	2.071	1.1587	1.1141	-0.3107	0.044564	BCC	BCC
PtPdCrScTa	7.6172	7.5539	6.0458	5.9991	0.06332	0.04669	4.51757	4.9104	-2.404	-2.478	-0.3928	0.074352	BCC	BCC
PtPdCrScRe	7.516	7.4559	5.9654	5.9511	0.06007	0.01433	7.90104	6.5842	2.2444	2.4396	1.31684	-0.19517	BCC	BCC
PtPdCrScHg	7.783	7.6703	6.1774	6.2132	0.11273	-0.03581	-0.6975	-0.541	1.4509	1.356	-0.1568	0.09492	FCC	FCC
PtPdCrScAl	7.565	7.6232	6.0044	5.8944	-0.0582	0.10995	-9.0278	-8.517	-5.5526	-5.498	-0.511	-0.05498	FCC	FCC
PtPdCrScGa	7.6126	7.695	6.0421	6.0333	-0.08242	0.00881	-7.1736	-9.197	-3.2603	-3.076	2.02332	-0.18455	FCC	FCC
PtPdCrScTl	7.9345	8.0509	6.2976	6.3086	-0.11644	-0.01101	5.35541	6.779	14.887	14.74	-1.4236	0.147399	FCC	FCC
PtPdCrScSi	7.6821	7.6203	6.0973	5.8741	0.06177	0.22317	-11.336	-11.45	-8.0509	-7.253	0.1145	-0.79784	FCC	FCC
PtPdCrScGe	7.7608	7.7365	6.1597	6.0383	0.02426	0.12142	-4.9079	-5.577	-0.9386	-1.031	0.66926	0.092826	FCC	FCC
PtPdCrScPb	7.984	8.0279	6.3369	6.3052	-0.04391	0.0317	4.5522	6.0696	9.7114	11.036	-1.5174	-1.32428	FCC	FCC
PtPdCrScP	7.7766	7.6367	6.1723	6.0062	0.13991	0.1661	-11.818	-11.47	-15.065	-15.69	-0.3442	0.627696	BCC	BCC
PtPdCrScAs	7.7269	7.6008	6.1328	5.9368	0.12608	0.19603	-7.5588	-9.332	-10.42	-9.738	1.77306	-0.68169	BCC	BCC
PtPdCrScSb	7.9812	7.9378	6.3347	6.2541	0.04338	0.08057	-2.3655	-2.297	5.4568	5.8675	-0.0689	-0.41072	FCC	FCC
PtPdCrTiV	7.2254	7.1993	5.7348	5.7716	0.02612	-0.03678	-11.623	-9.686	-7.5768	-7.216	-1.9372	-0.3608	FCC	FCC
PtPdCrTiMn	7.148	7.1543	5.6734	5.7187	-0.00629	-0.04532	-7.8505	-8.011	-2.7743	-3.083	0.16021	0.30825	FCC	FCC
PtPdCrTiZn	7.2774	7.2863	5.7761	5.803	-0.00888	-0.02691	-9.3522	-7.729	-10.738	-9.257	-1.6231	-1.48104	BCC	BCC

HEAs	Lattice parameters (Bohr)						ΔH_f (kJ/mol*f.u.)						Phase	
	FCC Cal.	FCC ML	BCC Cal.	BCC ML	FCC error	BCC error	FCC Cal.	FCC ML	BCC Cal.	BCC ML	FCC error	BCC error	Cal.	ML
PtPdCrTiY	7.7553	7.7712	6.1554	6.1426	-0.0159	0.01279	-5.11	-4.732	4.8941	5.1517	-0.3785	-0.25759	FCC	FCC
PtPdCrTiNb	7.3956	7.385	5.8699	5.8638	0.01056	0.00606	-8.6698	-7.347	-3.7658	-3.843	-1.3225	0.076854	FCC	FCC
PtPdCrTiTc	7.2834	7.2646	5.7808	5.8048	0.01879	-0.02397	-10.761	-10.25	-7.3622	-8.18	-0.5124	0.81802	FCC	FCC
PtPdCrTiCd	7.5344	7.4503	5.9801	6.0061	0.08414	-0.02601	-1.5121	-1.822	-2.9158	-2.751	0.30971	-0.16505	BCC	BCC
PtPdCrTiHf	7.5465	7.5215	5.9897	5.9926	0.02502	-0.00292	-6.39	-5.757	-8.8239	-9.488	-0.6332	0.664167	BCC	BCC
PtPdCrTiTa	7.4048	7.376	5.8772	5.8762	0.02883	0.00102	-4.0321	-3.804	-7.1496	-6.682	-0.2282	-0.46773	BCC	BCC
PtPdCrTiRe	7.2976	7.2687	5.7921	5.8261	0.02886	-0.03402	-4.6788	-4.293	-4.7355	-4.783	-0.3863	0.047833	BCC	BCC
PtPdCrTiHg	7.58	7.5622	6.0162	6.0994	0.01776	-0.08318	-6.6319	-7.209	-9.5894	-11.15	0.57669	1.56107	BCC	BCC
PtPdCrTiAl	7.3495	7.5285	5.8333	5.723	-0.17896	0.11034	-9.6927	-9.32	-9.1343	-9.134	-0.3728	0	FCC	FCC
PtPdCrTiGa	7.3999	7.5606	5.8733	5.8895	-0.16067	-0.01618	-9.8074	-9.081	-9.8956	-10.2	-0.7265	0.306051	BCC	BCC
PtPdCrTiTl	7.7394	7.7897	6.1427	6.172	-0.05033	-0.02926	4.5524	5.99	12.951	12.697	-1.4376	0.253948	FCC	FCC
PtPdCrTiSi	7.4734	7.5131	5.9317	5.7773	-0.03969	0.15435	-12.381	-10.96	-9.5475	-9.945	-1.4244	0.397812	FCC	FCC
PtPdCrTiGe	7.5565	7.5525	5.9976	5.9066	0.00398	0.09098	-10.72	-10.02	-11.43	-11.66	-0.7013	0.233272	BCC	BCC
PtPdCrTiPb	7.7914	7.7884	6.184	6.1854	0.003	-0.00136	4.94091	4.223	3.1329	3.4428	0.71791	-0.30985	BCC	BCC
PtPdCrTiP	7.5732	7.5056	6.0109	5.9135	0.06759	0.09735	-9.2796	-9.978	-10.968	-9.621	0.69847	-1.34697	BCC	FCC
PtPdCrTiAs	7.5207	7.4151	5.9692	5.8375	0.10563	0.1317	-9.468	-10.52	-8.678	-9.536	1.052	0.858267	FCC	FCC
PtPdCrTiSb	7.7885	7.7408	6.1817	6.1293	0.04765	0.0524	-5.3731	-5.268	-3.5042	-3.809	-0.1054	0.304712	FCC	FCC
PtPdCrVMn	7.0259	7.0384	5.5764	5.6342	-0.01254	-0.05777	-9.4705	-8.164	-5.1849	-5.034	-1.3063	-0.15102	FCC	FCC
PtPdCrVZn	7.1597	7.1767	5.6827	5.7134	-0.01701	-0.03075	-7.9876	-8.32	-8.023	-9.015	0.33282	0.991606	BCC	BCC
PtPdCrVY	7.6519	7.6509	6.0733	6.0586	0.00103	0.01474	-2.2184	-2.998	3.9484	3.6559	0.77943	0.292472	FCC	FCC
PtPdCrVNb	7.2817	7.2971	5.7795	5.7819	-0.01545	-0.00245	-8.898	-8.474	-3.1218	-3.321	-0.4237	0.199266	FCC	FCC
PtPdCrVTc	7.1659	7.162	5.6876	5.721	0.00386	-0.03345	-9.5297	-10.59	-8.3161	-9.344	1.05886	1.027829	FCC	FCC
PtPdCrVCd	7.4248	7.3455	5.8931	5.9195	0.07928	-0.02645	-1.6115	-1.31	-2.445	-2.243	-0.3013	-0.20188	BCC	BCC
PtPdCrVHf	7.4372	7.4312	5.9029	5.9181	0.00603	-0.01517	-6.6906	-5.719	-8.8043	-9.268	-0.9721	0.463385	BCC	BCC
PtPdCrVTa	7.2912	7.2839	5.787	5.7941	0.00731	-0.00706	-3.8966	-3.247	-6.8302	-6.044	-0.6494	-0.78577	BCC	BCC
PtPdCrVRe	7.1805	7.1672	5.6992	5.739	0.01329	-0.03984	-3.1957	-4.097	-5.3432	-6.213	0.90134	0.86982	BCC	BCC
PtPdCrVHg	7.4716	7.4881	5.9303	6.0143	-0.01646	-0.08405	-5.0896	-6.697	-10.997	-10.18	1.60726	-0.81462	BCC	BCC
PtPdCrVAl	7.2342	7.4329	5.7418	5.6421	-0.19873	0.09966	-9.6648	-9.293	-9.2311	-8.469	-0.3717	-0.7622	FCC	FCC

HEAs	Lattice parameters (Bohr)						ΔH_f (kJ/mol*f.u.)						Phase	
	FCC Cal.	FCC ML	BCC Cal.	BCC ML	FCC error	BCC error	FCC Cal.	FCC ML	BCC Cal.	BCC ML	FCC error	BCC error	Cal.	ML
PtPdCrVGa	7.2862	7.4575	5.783	5.817	-0.17135	-0.03398	-8.129	-8.557	-9.1494	-10.64	0.42784	1.489432	BCC	BCC
PtPdCrVTl	7.6356	7.6734	6.0604	6.0923	-0.03784	-0.03195	9.26436	7.532	9.2678	10.777	1.73236	-1.50871	FCC	FCC
PtPdCrVSi	7.3619	7.4819	5.8432	5.7164	-0.11999	0.12675	-10.465	-10.57	-8.8699	-8.87	0.1057	0	FCC	FCC
PtPdCrVGe	7.4475	7.4732	5.9111	5.8318	-0.02572	0.07927	-9.2909	-10.1	-12.259	-12.02	0.8079	-0.24038	BCC	BCC
PtPdCrVPb	7.689	7.7039	6.1028	6.1082	-0.0149	-0.00543	4.6469	4.1864	1.4259	1.455	0.4605	-0.0291	BCC	BCC
PtPdCrVP	7.4647	7.4454	5.9247	5.8585	0.01928	0.06622	-10.729	-10.03	-7.1251	-8.19	-0.7019	1.064674	FCC	FCC
PtPdCrVAs	7.4107	7.3167	5.8818	5.7723	0.09396	0.10954	-13.726	-10.89	-8.5279	-8.977	-2.8323	0.448835	FCC	FCC
PtPdCrVSb	7.686	7.6593	6.1004	6.0517	0.02667	0.04866	-4.3378	-5.784	-6.1134	-6.574	1.44593	0.460145	BCC	BCC
PtPdCrMnZn	7.0808	7.124	5.6201	5.642	-0.04318	-0.02195	-8.3047	-7.349	-6.1816	-7.025	-0.9554	0.84294	FCC	FCC
PtPdCrMnY	7.583	7.6148	6.0187	6.0184	-0.03177	0.00025	-2.6296	-2.529	4.4357	4.3918	-0.1011	0.043918	FCC	FCC
PtPdCrMnNb	7.2054	7.2276	5.719	5.7287	-0.02216	-0.00973	-2.7563	-2.702	-4.0711	-3.877	-0.054	-0.19386	BCC	BCC
PtPdCrMnTc	7.0871	7.0976	5.6251	5.6742	-0.01047	-0.04914	-9.5524	-8.529	-6.4714	-6.105	-1.0235	-0.36631	FCC	FCC
PtPdCrMnCd	7.3515	7.2999	5.8349	5.8723	0.05163	-0.03738	-0.1912	-0.22	-1.1696	-1.218	0.02857	0.048732	BCC	BCC
PtPdCrMnHf	7.3642	7.3691	5.845	5.8831	-0.00487	-0.03811	-2.5919	-2.541	-3.8421	-4.131	-0.0508	0.289191	BCC	BCC
PtPdCrMnTa	7.2152	7.2234	5.7267	5.7525	-0.00818	-0.02578	-0.4947	-0.43	-1.845	-1.708	-0.0645	-0.13666	BCC	BCC
PtPdCrMnRe	7.1021	7.1036	5.6369	5.7021	-0.00151	-0.06516	-1.6385	-1.546	-0.0515	-0.049	-0.0927	-0.00292	FCC	FCC
PtPdCrMnHg	7.3993	7.4338	5.8729	5.968	-0.03448	-0.09515	-4.4568	-5.065	-5.5113	-4.671	0.60774	-0.84071	BCC	FCC
PtPdCrMnAl	7.1569	7.3948	5.6805	5.6133	-0.23786	0.06717	-10.296	-9.276	-4.4777	-5.395	-1.0204	0.917116	FCC	FCC
PtPdCrMnGa	7.2101	7.4105	5.7226	5.7685	-0.20045	-0.04588	-10.711	-8.501	-9.388	-8.308	-2.2102	-1.08004	FCC	FCC
PtPdCrMnTl	7.5664	7.6626	6.0054	6.0453	-0.09624	-0.03988	5.68955	5.2681	9.4152	8.7178	0.42145	0.697424	FCC	FCC
PtPdCrMnSi	7.2874	7.3781	5.784	5.6799	-0.09071	0.10411	-9.3695	-9.968	-13.052	-12.92	0.59805	-0.12923	BCC	BCC
PtPdCrMnGe	7.3747	7.4166	5.8533	5.7934	-0.04192	0.05989	-10.995	-9.996	-9.123	-10.86	-0.9996	1.737712	FCC	BCC
PtPdCrMnPb	7.6208	7.6157	6.0486	6.0599	0.00507	-0.01129	5.9676	6.5578	-0.3681	-0.423	-0.5902	0.055003	BCC	BCC
PtPdCrMnP	7.3922	7.4271	5.8672	5.8271	-0.03488	0.04011	-9.394	-9.489	-8.8544	-9.624	0.09489	0.769944	FCC	BCC
PtPdCrMnAs	7.3371	7.2869	5.8235	5.7434	0.05023	0.08008	-7.8507	-9.129	-9.8662	-9.866	1.27802	0	BCC	BCC
PtPdCrMnSb	7.6177	7.5703	6.0462	6.007	0.04738	0.03916	-3.5156	-3.255	-4.3671	-5.02	-0.2604	0.652548	BCC	BCC
PtPdCrZnY	7.6983	7.6297	6.1102	6.1052	0.06864	0.00498	-7.2261	-6.339	-4.5002	-3.814	-0.8874	-0.68647	FCC	FCC
PtPdCrZnNb	7.3329	7.313	5.8201	5.8086	0.01985	0.01149	-8.3546	-8.272	-9.1909	-8.134	-0.0827	-1.05736	BCC	FCC

HEAs	Lattice parameters (Bohr)						ΔH_f (kJ/mol*f.u.)						Phase	
	FCC Cal.	FCC ML	BCC Cal.	BCC ML	FCC error	BCC error	FCC Cal.	FCC ML	BCC Cal.	BCC ML	FCC error	BCC error	Cal.	ML
PtPdCrZnTc	7.2187	7.2193	5.7295	5.7445	-0.00058	-0.015	-9.5214	-8.735	-8.9133	-9.904	-0.7862	0.99037	FCC	BCC
PtPdCrZnCd	7.4741	7.4367	5.9322	5.9361	0.03735	-0.00394	-6.5392	-7.693	-3.5505	-3.481	1.15398	-0.06962	FCC	FCC
PtPdCrZnHf	7.4863	7.459	5.9419	5.9429	0.02733	-0.00099	-5.1989	-5.305	-4.4108	-3.903	0.1061	-0.50744	FCC	FCC
PtPdCrZnTa	7.3423	7.3141	5.8276	5.8269	0.02819	0.00068	-6.1309	-6.321	-2.8159	-2.873	0.18962	0.057468	FCC	FCC
PtPdCrZnRe	7.2331	7.2113	5.741	5.7738	0.02184	-0.03285	-2.8323	-3.497	-2.539	-2.189	0.66437	-0.35021	FCC	FCC
PtPdCrZnHg	7.5203	7.5349	5.9689	6.023	-0.0146	-0.05413	-9.0907	-8.417	-2.258	-2.11	-0.6734	-0.14772	FCC	FCC
PtPdCrZnAl	7.286	7.402	5.7829	5.7217	-0.11596	0.06123	-9.6914	-9.23	-9.2608	-10.77	-0.4615	1.507576	FCC	BCC
PtPdCrZnGa	7.3373	7.4735	5.8236	5.8621	-0.1362	-0.03848	-7.1583	-9.544	-11.155	-12.26	2.3861	1.103283	BCC	BCC
PtPdCrZnTl	7.6822	7.6178	6.0974	6.1096	0.06438	-0.01225	4.67678	5.0288	1.7036	1.8929	-0.352	-0.18929	BCC	BCC
PtPdCrZnSi	7.412	7.3263	5.8829	5.7742	0.08572	0.10872	-10.233	-10.13	-14.403	-15.32	-0.1013	0.919338	BCC	BCC
PtPdCrZnGe	7.4965	7.4036	5.9499	5.8786	0.09285	0.07134	-9.1706	-10.3	-14.249	-14.25	1.13344	0	BCC	BCC
PtPdCrZnPb	7.735	7.6097	6.1393	6.131	0.12528	0.00826	-1.7304	-1.531	-2.9762	-2.588	-0.1991	-0.3882	BCC	BCC
PtPdCrZnP	7.5134	7.4426	5.9634	5.8598	0.07083	0.10361	-8.4634	-8.816	-9.2759	-9.184	0.35264	-0.09184	BCC	BCC
PtPdCrZnAs	7.4601	7.3356	5.9211	5.7948	0.12452	0.1263	-9.9687	-9.404	-7.8078	-7.58	-0.5643	-0.22741	FCC	FCC
PtPdCrZnSb	7.732	7.6003	6.1369	6.0771	0.13168	0.05978	-6.6997	-5.63	-7.2771	-7.426	-1.0697	0.148512	BCC	BCC
PtPdCrYNb	7.8042	7.7761	6.1942	6.1869	0.02808	0.00728	-7.5364	-6.851	5.4772	5.2164	-0.6851	0.26082	FCC	FCC
PtPdCrYTc	7.7037	7.7311	6.1144	6.1058	-0.02741	0.00862	0.48937	0.5262	1.2277	1.3794	-0.0368	-0.15173	FCC	FCC
PtPdCrYCd	7.9292	7.9032	6.2934	6.2885	0.02604	0.00494	-1.2693	-1.113	3.776	3.4963	-0.1559	0.279704	FCC	FCC
PtPdCrYHf	7.9402	7.9342	6.3021	6.3047	0.00596	-0.00259	-0.5886	-0.626	5.1974	4.8574	0.03757	0.340018	FCC	FCC
PtPdCrYTa	7.8125	7.8136	6.2008	6.1932	-0.00109	0.00759	-5.8929	-5.124	4.8442	4.4854	-0.7686	0.358832	FCC	FCC
PtPdCrYRe	7.7164	7.761	6.1245	6.1385	-0.04465	-0.01403	0.01423	0.0153	2.7586	2.6525	-0.0011	0.1061	FCC	FCC
PtPdCrYHg	7.9704	8.0447	6.3261	6.3813	-0.07432	-0.05521	-2.1576	-2.397	-1.5422	-1.59	0.23973	0.047697	FCC	FCC
PtPdCrYAl	7.7629	7.7544	6.1614	6.1377	0.0085	0.02371	-2.998	-2.968	-8.4905	-8.164	-0.0297	-0.32656	BCC	BCC
PtPdCrYGa	7.8081	7.9065	6.1973	6.2259	-0.0984	-0.0286	-3.0626	-2.47	-4.8309	-5.195	-0.5928	0.363615	BCC	BCC
PtPdCrYTl	7.6356	7.9356	6.4409	6.4151	-0.3	0.02575	-0.8894	-0.754	16.029	16.191	-0.1357	-0.16191	FCC	FCC
PtPdCrYSi	7.8742	7.8002	6.2498	6.1509	0.074	0.09886	-8.144	-8.144	-8.8543	-8.767	0	-0.08767	BCC	BCC
PtPdCrYGe	7.9492	7.9416	6.3093	6.2649	0.00755	0.04435	-2.0757	-2.306	-4.5147	-4.752	0.23063	0.237615	BCC	BCC
PtPdCrYPb	8.1623	8.223	6.4785	6.3769	-0.06066	0.10155	-4.5384	-4.322	11.621	12.106	-0.2161	-0.48422	FCC	FCC

HEAs	Lattice parameters (Bohr)						ΔH_f (kJ/mol*f.u.)						Phase	
	FCC Cal.	FCC ML	BCC Cal.	BCC ML	FCC error	BCC error	FCC Cal.	FCC ML	BCC Cal.	BCC ML	FCC error	BCC error	Cal.	ML
PtPdCrYP	7.9643	7.7924	6.3212	6.2817	0.17186	0.03953	-13.384	-14.24	-14.692	-15.63	0.8543	0.937812	BCC	BCC
PtPdCrYAs	7.9169	7.7615	6.2836	6.2189	0.15536	0.06472	-13.095	-10.48	-7.8185	-9.653	-2.6191	1.833975	FCC	FCC
PtPdCrYSb	8.1596	8.2168	6.4763	6.3714	-0.05716	0.10491	-4.4566	-4.204	5.1891	5.0874	-0.2523	0.101748	FCC	FCC
PtPdCrNbTc	7.3387	7.3474	5.8248	5.8197	-0.00866	0.00506	-4.8284	-5.961	-6.8401	-5.948	1.13259	-0.89219	BCC	FCC
PtPdCrNbCd	7.5862	7.54	6.0212	6.0072	0.04619	0.01397	-1.7997	-2.117	-3.5993	-4.044	0.3176	0.444862	BCC	BCC
PtPdCrNbHf	7.5981	7.5792	6.0306	6.0119	0.01892	0.01873	-3.1857	-2.723	-3.7897	-4.119	-0.4629	0.329536	BCC	BCC
PtPdCrNbTa	7.4584	7.4422	5.9197	5.8954	0.0162	0.02433	-3.1759	-4.292	-10.014	-11.25	1.11587	1.237676	BCC	BCC
PtPdCrNbRe	7.3527	7.3527	5.8358	5.8377	-1E-05	-0.00186	2.38571	2.0048	-2.648	-2.364	0.38091	-0.28372	BCC	BCC
PtPdCrNbHg	7.6311	7.6744	6.0568	6.0924	-0.0433	-0.03559	-1.5516	-1.825	-4.4719	-4.809	0.27381	0.336595	BCC	BCC
PtPdCrNbAl	7.4039	7.4177	5.8765	5.7528	-0.01379	0.12368	-12.244	-12.37	-8.0732	-7.02	0.12368	-1.05303	FCC	FCC
PtPdCrNbGa	7.4536	7.5622	5.9159	5.9284	-0.10864	-0.01251	-11.92	-10.36	-11.359	-12.91	-1.5547	1.5489	FCC	BCC
PtPdCrNbTl	7.7885	7.8129	6.1817	6.1908	-0.02445	-0.0091	4.99928	5.1539	10.519	10.213	-0.1546	0.306375	FCC	FCC
PtPdCrNbSi	7.526	7.4944	5.9734	5.8065	0.03161	0.16689	-10.924	-13.49	-8.8042	-7.932	2.56251	-0.87249	FCC	FCC
PtPdCrNbGe	7.6079	7.5443	6.0384	5.9445	0.06364	0.09393	-15.787	-12.83	-7.3184	-7.955	-2.952	0.636384	FCC	FCC
PtPdCrNbPb	7.8398	7.8665	6.2225	6.2029	-0.02667	0.01958	2.74849	3.4791	5.3992	5.8056	-0.7306	-0.40639	FCC	FCC
PtPdCrNbP	7.6244	7.4981	6.0515	5.9672	0.12632	0.08431	-8.0535	-10.74	-10.885	-10.27	2.6845	-0.61613	BCC	FCC
PtPdCrNbAs	7.5727	7.3825	6.0104	5.8767	0.19017	0.13373	-9.776	-11.37	-8.3428	-8.022	1.59144	-0.32088	FCC	FCC
PtPdCrNbSb	7.8369	7.7976	6.2202	6.1436	0.03932	0.07656	-1.8821	-1.981	-3.5377	-3.369	0.09906	-0.16846	BCC	BCC
PtPdCrTcCd	7.4797	7.4039	5.9367	5.9546	0.07582	-0.01794	-1.0628	-0.864	-1.9171	-2.154	-0.1987	0.23694	BCC	BCC
PtPdCrTcHf	7.492	7.4873	5.9464	5.956	0.00468	-0.00961	-4.4415	-4.579	-7.9204	-7.543	0.13737	-0.37716	BCC	BCC
PtPdCrTcTa	7.3482	7.3433	5.8322	5.8335	0.00486	-0.00126	-0.5056	-0.665	-3.9034	-4.386	0.15967	0.482438	BCC	BCC
PtPdCrTcRe	7.2392	7.2264	5.7458	5.7795	0.01279	-0.03375	-2.1242	-2.284	-5.7339	-4.943	0.15989	-0.79088	BCC	BCC
PtPdCrTcHg	7.5259	7.5288	5.9733	6.0454	-0.0029	-0.07209	-6.39	-5.809	-8.3683	-7.277	-0.5809	-1.09152	BCC	BCC
PtPdCrTcAl	7.292	7.3942	5.7877	5.7033	-0.1022	0.08436	-9.5914	-10.43	-8.4189	-8.095	0.83403	-0.3238	FCC	FCC
PtPdCrTcGa	7.3432	7.4836	5.8283	5.8725	-0.14043	-0.04422	-6.88	-8.709	-11.817	-10.84	1.82887	-0.97568	BCC	BCC
PtPdCrTcTl	7.6875	7.7245	6.1016	6.123	-0.03696	-0.0214	7.31811	8.2226	5.6525	6.3511	-0.9045	-0.69862	BCC	BCC
PtPdCrTcSi	7.4178	7.4076	5.8875	5.7726	0.01018	0.1149	-12.483	-11.78	-8.3742	-8.291	-0.7066	-0.08291	FCC	FCC
PtPdCrTcGe	7.5021	7.464	5.9544	5.8991	0.03808	0.05531	-11.251	-10.72	-9.6047	-10.33	-0.5358	0.722932	FCC	FCC

HEAs	Lattice parameters (Bohr)						ΔH_f (kJ/mol*f.u.)						Phase	
	FCC Cal.	FCC ML	BCC Cal.	BCC ML	FCC error	BCC error	FCC Cal.	FCC ML	BCC Cal.	BCC ML	FCC error	BCC error	Cal.	ML
PtPdCrTcPb	7.7403	7.7423	6.1435	6.1417	-0.00203	0.00175	5.92745	4.7802	-2.3863	-2.681	1.14725	0.294932	BCC	BCC
PtPdCrTcP	7.519	7.4545	5.9679	5.8967	0.06454	0.07116	-9.1847	-8.917	-7.4632	-7.856	-0.2675	0.3928	FCC	FCC
PtPdCrTcAs	7.4658	7.3192	5.9256	5.8173	0.1466	0.10831	-12.555	-10.12	-8.1213	-8.203	-2.43	0.082033	FCC	FCC
PtPdCrTcSb	7.7373	7.6843	6.1411	6.0995	0.05297	0.04158	-6.0586	-7.045	-8.8152	-7.871	0.98629	-0.94448	BCC	BCC
PtPdCrCdHf	7.7299	7.6444	6.1352	6.1329	0.08548	0.00231	-1.7939	-2.039	-3.2357	-2.789	0.24462	-0.4463	BCC	BCC
PtPdCrCdTa	7.595	7.5183	6.0282	6.0182	0.07671	0.00996	-0.3313	-0.286	-2.3057	-2.681	-0.0457	0.375354	BCC	BCC
PtPdCrCdRe	7.4932	7.3809	5.9473	5.9787	0.11225	-0.03138	5.89388	5.3098	0.7661	0.912	0.58408	-0.14592	BCC	BCC
PtPdCrCdHg	7.7618	7.6957	6.1605	6.2202	0.06606	-0.05969	1.33047	1.3173	-0.1384	-0.127	0.01317	-0.01143	BCC	BCC
PtPdCrCdAl	7.5425	7.5067	5.9865	5.9106	0.03578	0.07587	-8.7815	-7.57	-6.4814	-6.895	-1.2112	0.413706	FCC	FCC
PtPdCrCdGa	7.5904	7.6168	6.0245	6.0479	-0.02645	-0.02344	-7.827	-8.997	-8.7595	-8.036	1.16956	-0.72326	BCC	FCC
PtPdCrCdTl	7.914	7.8788	6.2814	6.2818	0.0352	-0.00045	7.45867	7.1718	7.9597	7.1069	0.28687	0.852828	FCC	BCC
PtPdCrCdSi	7.6602	7.4724	6.0799	5.9384	0.18784	0.14154	-8.6464	-10.81	-12.143	-10.65	2.1616	-1.49124	BCC	FCC
PtPdCrCdGe	7.7394	7.5899	6.1427	6.0764	0.14947	0.06634	-5.402	-4.657	-5.4733	-5.419	-0.7451	-0.05419	BCC	BCC
PtPdCrCdPb	7.9638	7.8494	6.3209	6.295	0.11438	0.02586	5.13834	5.2432	0.9284	0.8073	-0.1049	0.121095	BCC	BCC
PtPdCrCdP	7.7553	7.5716	6.1554	6.0147	0.1837	0.14069	-8.5108	-9.456	-9.946	-8.574	0.94564	-1.37186	BCC	FCC
PtPdCrCdAs	7.7053	7.5605	6.1157	5.9601	0.1448	0.1556	-11.271	-9.163	-8.3585	-7.397	-2.1075	-0.9616	FCC	FCC
PtPdCrCdSb	7.961	7.7854	6.3186	6.2624	0.17556	0.05622	-1.4434	-1.266	-4.0054	-3.366	-0.1773	-0.63952	BCC	BCC
PtPdCrHfTa	7.6069	7.5693	6.0376	6.0281	0.03761	0.00951	-1.0017	-0.973	-9.1868	-9.471	-0.0292	0.284127	BCC	BCC
PtPdCrHfRe	7.5054	7.483	5.957	5.9912	0.02237	-0.03418	-1.7457	-1.695	-3.1789	-3.532	-0.0508	0.35321	BCC	BCC
PtPdCrHfHg	7.7732	7.746	6.1696	6.224	0.02715	-0.05445	-1.4863	-1.416	-3.4571	-3.799	-0.0708	0.34191	BCC	BCC
PtPdCrHfAl	7.5545	7.5503	5.9961	5.9375	0.00424	0.05855	-12.81	-11.86	-6.3894	-6.945	-0.9489	0.5556	FCC	FCC
PtPdCrHfGa	7.6023	7.6804	6.0339	6.0807	-0.07814	-0.04679	-12.392	-10.78	-15.01	-14.57	-1.6163	-0.4372	BCC	BCC
PtPdCrHfTl	7.925	7.9631	6.29	6.3288	-0.03814	-0.03876	5.61568	5.1997	17.994	16.066	0.41598	1.927896	FCC	FCC
PtPdCrHfSi	7.6719	7.5692	6.0892	5.9639	0.10273	0.12532	-11.072	-14.96	-7.4862	-6.806	3.89012	-0.68056	FCC	FCC
PtPdCrHfGe	7.7508	7.6819	6.1518	6.1074	0.06893	0.04444	-13.582	-12.02	-9.7248	-9.004	-1.5625	-0.72035	FCC	FCC
PtPdCrHfPb	7.9746	7.9719	6.3295	6.3195	0.00271	0.00995	2.70025	2.3278	8.7711	8.7711	0.37245	0	FCC	FCC
PtPdCrHfP	7.7667	7.5805	6.1644	6.1182	0.18621	0.04624	-10.642	-10.33	-9.3642	-8.143	-0.31	-1.22142	FCC	FCC
PtPdCrHfAs	7.7169	7.5267	6.1249	6.0378	0.19015	0.08707	-11.594	-10.94	-5.7866	-6.651	-0.6562	0.864669	FCC	FCC

HEAs	Lattice parameters (Bohr)						ΔH_f (kJ/mol*f.u.)						Phase	
	FCC Cal.	FCC ML	BCC Cal.	BCC ML	FCC error	BCC error	FCC Cal.	FCC ML	BCC Cal.	BCC ML	FCC error	BCC error	Cal.	ML
PtPdCrHfSb	7.9718	7.9206	6.3272	6.2797	0.05119	0.04751	-3.8102	-3.496	-3.0697	-3.337	-0.3146	0.266928	FCC	FCC
PtPdCrTaRe	7.3621	7.3535	5.8433	5.8598	0.00858	-0.01651	1.36392	1.1366	-2.0612	-2.316	0.22732	0.25476	BCC	BCC
PtPdCrTaHg	7.6398	7.6247	6.0637	6.106	0.01511	-0.04228	-1.1567	-0.89	-2.1071	-2.15	-0.2669	0.043002	BCC	BCC
PtPdCrTaAl	7.4132	7.4016	5.8838	5.7887	0.01156	0.09513	-14.038	-13.5	-6.2088	-6.087	-0.5399	-0.12174	FCC	FCC
PtPdCrTaGa	7.4627	7.5215	5.9231	5.9643	-0.05881	-0.04116	-11.998	-10.91	-11.688	-13.28	-1.0907	1.593828	FCC	BCC
PtPdCrTaTl	7.7968	7.8034	6.1883	6.2041	-0.00659	-0.01576	4.36136	4.0383	19.386	18.118	0.32306	1.268274	FCC	FCC
PtPdCrTaSi	7.535	7.4808	5.9805	5.8655	0.05416	0.115	-16.516	-14.36	-6.2233	-6.993	-2.1542	0.769175	FCC	FCC
PtPdCrTaGe	7.6167	7.535	6.0454	5.9872	0.08171	0.05818	-16.226	-13.75	-8.4839	-8.93	-2.4751	0.44652	FCC	FCC
PtPdCrTaPb	7.8481	7.8316	6.229	6.2192	0.01649	0.00983	5.29402	4.8569	12.448	11.634	0.43712	0.814359	FCC	FCC
PtPdCrTaP	7.6332	7.4754	6.0584	6.0389	0.15775	0.01954	-8.338	-9.264	-6.9918	-7.769	0.92644	0.77687	FCC	FCC
PtPdCrTaAs	7.5815	7.3746	6.0175	5.9503	0.20692	0.06715	-7.5203	-10.03	-7.2374	-7.166	2.50678	-0.07166	FCC	FCC
PtPdCrTaSb	7.8452	7.7873	6.2267	6.1749	0.05788	0.05182	-2.7386	-2.536	-7.9883	-8.876	-0.2029	0.88759	BCC	BCC
PtPdCrReHg	7.5392	7.5	5.9838	6.065	0.03917	-0.08116	3.22134	3.039	1.4228	1.3949	0.18234	0.027898	BCC	BCC
PtPdCrReAl	7.3061	7.3483	5.7989	5.727	-0.04216	0.07188	-9.7811	-10.87	-6.916	-6.715	1.08679	-0.20144	FCC	FCC
PtPdCrReGa	7.3571	7.4517	5.8393	5.8898	-0.09459	-0.05046	-10.037	-10.46	-9.3197	-10.24	0.4182	0.921726	FCC	FCC
PtPdCrReTl	7.7003	7.7162	6.1117	6.1367	-0.01594	-0.025	9.04067	7.5972	10.409	11.565	1.44347	-1.15654	FCC	FCC
PtPdCrReSi	7.4314	7.3994	5.8983	5.8001	0.03204	0.09824	-10.266	-12.52	-8.0345	-7.238	2.2536	-0.79621	FCC	FCC
PtPdCrReGe	7.5154	7.454	5.965	5.9287	0.06144	0.03631	-13.843	-11.35	-8.9449	-8.77	-2.4963	-0.17539	FCC	FCC
PtPdCrRePb	7.7528	7.7229	6.1534	6.1551	0.02992	-0.00169	6.33251	6.6658	1.036	1.036	-0.3333	0	BCC	BCC
PtPdCrReP	7.5323	7.4474	5.9784	5.9357	0.08493	0.04272	-7.6121	-8.65	-6.282	-6.613	1.03801	0.33063	FCC	FCC
PtPdCrReAs	7.4793	7.3273	5.9363	5.8644	0.15199	0.07192	-7.7363	-8.319	-7.9201	-7.072	0.5823	-0.84858	BCC	FCC
PtPdCrReSb	7.7498	7.6622	6.1511	6.1215	0.08763	0.02955	-0.0332	-0.031	-0.8915	-0.841	-0.0027	-0.05046	BCC	BCC
PtPdCrHgAl	7.5879	7.5539	6.0225	6.017	0.03401	0.00553	-8.0532	-8.218	-6.4732	-7.356	0.16435	0.882708	FCC	FCC
PtPdCrHgGa	7.6352	7.6983	6.0601	6.1452	-0.0631	-0.08513	-8.6246	-9.175	-13.323	-11.69	0.55051	-1.63612	BCC	BCC
PtPdCrHgTl	7.9553	7.9624	6.3141	6.3689	-0.00711	-0.05478	5.44555	6.8931	6.1963	6.7351	-1.4476	-0.53881	FCC	BCC
PtPdCrHgSi	7.7043	7.5257	6.1149	6.0485	0.17859	0.0664	-12.371	-12.13	-10.988	-9.899	-0.2426	-1.08892	FCC	FCC
PtPdCrHgGe	7.7825	7.6571	6.177	6.1703	0.12543	0.0067	-8.9864	-9.459	-7.961	-7.729	0.47297	-0.23187	FCC	FCC
PtPdCrHgPb	8.0046	7.9964	6.3532	6.4001	0.00817	-0.04687	3.52869	4.4667	-2.003	-1.945	-0.938	-0.05834	BCC	BCC

HEAs	Lattice parameters (Bohr)						ΔH_f (kJ/mol*f.u.)						Phase	
	FCC Cal.	FCC ML	BCC Cal.	BCC ML	FCC error	BCC error	FCC Cal.	FCC ML	BCC Cal.	BCC ML	FCC error	BCC error	Cal.	ML
PtPdCrHgP	7.7983	7.6226	6.1895	6.0969	0.17569	0.09261	-6.9663	-8.708	-7.072	-7.216	1.74158	0.144326	BCC	FCC
PtPdCrHgAs	7.7488	7.5913	6.1503	6.0435	0.15754	0.10676	-11.376	-11.61	-8.6984	-7.836	0.23216	-0.862	FCC	FCC
PtPdCrHgSb	8.0018	7.8501	6.351	6.3457	0.15167	0.00531	-3.1628	-3.071	2.3401	2.2501	-0.0921	0.090004	FCC	FCC
PtPdCrAlGa	7.7083	7.8587	5.8799	5.8433	-0.15043	0.03664	-8.1079	-9.539	-10.241	-9.144	1.43081	-1.09728	BCC	FCC
PtPdCrAlTl	7.747	7.8178	6.1488	6.1114	-0.0708	0.03739	-1.3156	-1.709	-7.7239	-7.427	0.39298	-0.29707	BCC	BCC
PtPdCrAlSi	7.4816	7.7775	5.9381	5.7046	-0.29591	0.23354	-10.998	-10.78	-14.41	-12.64	-0.2156	-1.76968	BCC	BCC
PtPdCrAlGe	7.5645	7.7284	6.0039	5.8017	-0.16392	0.20223	-8.8383	-10.28	-11.38	-11.05	1.43879	-0.33147	BCC	BCC
PtPdCrAlPb	7.7989	7.9207	6.19	6.1285	-0.12177	0.06151	-2.3268	-2.237	-9.609	-11.17	-0.0895	1.564262	BCC	BCC
PtPdCrAlP	7.5812	7.6088	6.0172	5.8289	-0.02765	0.18827	-8.7351	-9.099	-9.6404	-9.451	0.36396	-0.18903	BCC	BCC
PtPdCrAlAs	7.5288	7.4733	5.9756	5.766	0.0555	0.20961	-12.486	-10.41	-9.6835	-10.19	-2.0811	0.50966	FCC	FCC
PtPdCrAlSb	7.796	7.8169	6.1877	6.0135	-0.02092	0.17417	-12.087	-9.443	-8.749	-8.254	-2.6441	-0.49523	FCC	FCC
PtPdCrGaTl	7.7924	7.9023	6.1848	6.2368	-0.10991	-0.05198	-0.3824	-0.407	-3.1477	-2.786	0.02441	-0.36213	BCC	BCC
PtPdCrGaSi	7.5302	7.6863	5.9767	5.8471	-0.15608	0.12964	-9.1499	-10.17	-12.074	-12.45	1.01666	0.373422	BCC	BCC
PtPdCrGaGe	7.6121	7.7294	6.0417	5.9767	-0.11733	0.065	-9.0855	-10.56	-13.467	-13.88	1.47903	0.41652	BCC	BCC
PtPdCrGaPb	7.8437	7.952	6.2256	6.2435	-0.10828	-0.01793	-3.3044	-2.777	-9.8164	-8.611	-0.5276	-1.20553	BCC	BCC
PtPdCrGaP	7.6285	7.6335	6.0548	5.9393	-0.00497	0.11547	-10.847	-9.271	-9.9777	-8.909	-1.5761	-1.06904	FCC	FCC
PtPdCrGaAs	7.5768	7.5493	6.0137	5.9032	0.02754	0.11054	-10.179	-10.28	-11.052	-9.957	0.10281	-1.09529	BCC	FCC
PtPdCrGaSb	7.8408	7.9144	6.2233	6.177	-0.07359	0.04625	-6.3001	-8.75	-12.602	-11.56	2.45003	-1.0405	BCC	BCC
PtPdCrTlSi	7.8588	7.8772	6.2375	6.112	-0.01845	0.12549	-1.1671	-0.934	-7.8395	-8.711	-0.2334	0.87106	BCC	BCC
PtPdCrTlGe	7.934	7.9436	6.2972	6.2403	-0.00961	0.05691	1.27658	1.5568	-2.6911	-2.492	-0.2802	-0.19934	BCC	BCC
PtPdCrTlPb	8.148	8.1607	6.467	6.5004	-0.01274	-0.03336	7.56925	8.905	6.5444	7.037	-1.3358	-0.49259	BCC	BCC
PtPdCrTlP	7.9492	7.8715	6.3093	6.1811	0.07765	0.12815	-10.249	-8.401	-10.933	-9.85	-1.8482	-1.08348	BCC	BCC
PtPdCrTlAs	7.9016	7.8015	6.2715	6.1405	0.10008	0.13099	-2.9335	-3.667	-3.9519	-3.407	0.73338	-0.54509	BCC	FCC
PtPdCrTlSb	8.1453	8.1359	6.4649	6.4111	0.00936	0.05379	4.74871	4.6104	3.6996	3.9357	0.13831	-0.23614	BCC	BCC
PtPdCrSiGe	7.6816	7.6683	6.0969	5.8027	0.01327	0.29416	-12.067	-11.28	-16.034	-14.71	-0.7895	-1.32391	BCC	BCC
PtPdCrSiPb	7.9092	7.9525	6.2776	6.1528	-0.04327	0.12476	-4.1928	-5.375	-11.491	-10.26	1.18259	-1.2312	BCC	BCC
PtPdCrSiP	7.6977	7.6087	6.1097	5.7852	0.08904	0.3245	-8.134	-7.821	-9.0222	-8.128	-0.3128	-0.89409	BCC	BCC
PtPdCrSiAs	7.647	7.4816	6.0694	5.7435	0.16538	0.32591	-8.6746	-9.533	-8.6588	-8.573	0.85793	-0.08573	FCC	FCC

HEAs	Lattice parameters (Bohr)						ΔH_f (kJ/mol*f.u.)						Phase	
	FCC Cal.	FCC ML	BCC Cal.	BCC ML	FCC error	BCC error	FCC Cal.	FCC ML	BCC Cal.	BCC ML	FCC error	BCC error	Cal.	ML
PtPdCrSiSb	7.9064	7.7692	6.2753	5.9963	0.13716	0.27898	-9.8727	-11.35	-17.073	-14.98	1.47523	-2.09672	BCC	BCC
PtPdCrGePb	7.9835	8.0476	6.3365	6.2771	-0.06408	0.05943	-0.913	-0.87	-11.527	-9.852	-0.0435	-1.67484	BCC	BCC
PtPdCrGeP	7.7761	7.6745	6.1719	5.8364	0.10161	0.33551	-10.557	-8.184	-7.2601	-8.345	-2.3732	1.084837	FCC	BCC
PtPdCrGeAs	7.7264	7.5492	6.1324	5.8053	0.17718	0.32713	-13.511	-10.56	-11.164	-10.84	-2.9556	-0.32516	FCC	BCC
PtPdCrGeSb	7.9807	7.9081	6.3343	6.1694	0.07261	0.16489	-9.486	-9.486	-7.8297	-8.156	0	0.326236	FCC	FCC
PtPdCrPbP	7.9985	7.9851	6.3484	6.2132	0.0134	0.13521	-8.4681	-9.963	-12.565	-11.63	1.49438	-0.9307	BCC	BCC
PtPdCrPbAs	7.9515	7.9447	6.3111	6.1886	0.00682	0.12252	-5.9909	-7.489	-9.7701	-8.723	1.49772	-1.0468	BCC	BCC
PtPdCrPbSb	8.1923	8.2675	6.5022	6.442	-0.07522	0.06022	4.16245	4.5244	-1.8188	-2.021	-0.362	0.20209	BCC	BCC
PtPdCrPAs	7.7424	7.6244	6.1451	5.7495	0.11796	0.39562	-10.264	-9.247	-7.4608	-7.244	-1.0172	-0.21731	FCC	FCC
PtPdCrPSb	7.9957	7.86	6.3462	6.0392	0.1357	0.30699	-9.6994	-9.797	-12.552	-13.08	0.09797	0.523016	BCC	BCC
PtPdCrAsSb	7.9487	7.8218	6.3089	5.9985	0.12688	0.31037	-12.809	-11.54	-13.167	-14.47	-1.2693	1.302273	BCC	BCC
PtPdNiSnSc	7.8726	7.837	6.2485	6.2236	0.03556	0.02486	-1.1704	-0.967	6.2509	6.0688	-0.2031	0.182064	FCC	FCC
PtPdNiSnTi	7.6743	7.6707	6.0911	6.1028	0.00357	-0.01173	-2.4526	-3.105	-1.7285	-1.82	0.65197	0.090975	FCC	FCC
PtPdNiSnV	7.5687	7.5729	6.0073	6.025	-0.00424	-0.01775	-3.2696	-3.892	-3.7549	-3.323	0.62278	-0.43198	BCC	FCC
PtPdNiSnMn	7.4982	7.5276	5.9513	5.9648	-0.02939	-0.01347	-1.3188	-1.127	-0.9138	-0.914	-0.1916	0	FCC	FCC
PtPdNiSnZn	7.6161	7.6127	6.0449	6.0295	0.00339	0.01539	-3.284	-3.252	-2.176	-2.365	-0.0325	0.189216	FCC	FCC
PtPdNiSnY	8.0558	8.0583	6.3939	6.378	-0.00248	0.01591	-3.846	-3.127	3.1383	2.7054	-0.7192	0.432864	FCC	FCC
PtPdNiSnNb	7.7242	7.6932	6.1307	6.1198	0.03097	0.01088	0.55744	0.4532	-2.3809	-2.357	0.10424	-0.02357	BCC	BCC
PtPdNiSnTc	7.6215	7.6184	6.0492	6.0594	0.00314	-0.01018	-3.7094	-3.533	-6.1605	-5.452	-0.1766	-0.70873	BCC	BCC
PtPdNiSnCd	7.8518	7.795	6.232	6.2239	0.05678	0.00806	2.06004	1.7458	1.8789	1.8066	0.31424	0.072264	BCC	FCC
PtPdNiSnHf	7.8629	7.8512	6.2408	6.2374	0.01171	0.00339	-1.3887	-1.543	-1.9155	-2.152	0.1543	0.236753	BCC	BCC
PtPdNiSnTa	7.7327	7.7106	6.1374	6.1294	0.02208	0.00803	-0.7283	-0.751	-6.5168	-5.667	0.02252	-0.85002	BCC	BCC
PtPdNiSnRe	7.6345	7.6224	6.0595	6.0754	0.01209	-0.0159	1.34895	1.173	1.6211	1.7621	0.17595	-0.14097	FCC	FCC
PtPdNiSnHg	7.8937	7.8843	6.2653	6.2986	0.00942	-0.03335	0.80235	0.6631	0.0038	0.0039	0.13925	-7.8E-05	BCC	BCC
PtPdNiSnAl	7.682	7.746	6.0972	5.9736	-0.06398	0.12363	-7.8158	-7.041	-8.4925	-9.542	-0.7745	1.049631	BCC	BCC
PtPdNiSnGa	7.7282	7.8115	6.1339	6.1263	-0.08332	0.00756	-6.4901	-6.979	-10.987	-11.81	0.4885	0.826959	BCC	BCC
PtPdNiSnTl	8.0411	7.9697	6.3822	6.385	0.07136	-0.00281	5.74226	6.7556	5.6371	5.4729	-1.0133	0.164187	BCC	BCC
PtPdNiSnSi	7.7956	7.7297	6.1874	5.9399	0.06593	0.2475	-13.068	-11.36	-18.179	-17.82	-1.7045	-0.35644	BCC	BCC

HEAs	Lattice parameters (Bohr)						ΔH_f (kJ/mol*f.u.)						Phase	
	FCC Cal.	FCC ML	BCC Cal.	BCC ML	FCC error	BCC error	FCC Cal.	FCC ML	BCC Cal.	BCC ML	FCC error	BCC error	Cal.	ML
PtPdNiSnGe	7.8721	7.8292	6.2481	6.1194	0.04288	0.12867	-8.1541	-10.32	-10.27	-11.16	2.16754	0.893048	BCC	BCC
PtPdNiSnPb	8.0893	8.087	6.4205	6.4332	0.00229	-0.01272	5.72193	6.3577	-0.6061	-0.645	-0.6358	0.038688	BCC	BCC
PtPdNiSnP	7.8875	7.8323	6.2603	5.9448	0.05518	0.3155	-13.076	-12.22	-9.4161	-8.639	-0.8554	-0.77747	FCC	FCC
PtPdNiSnAs	7.8392	7.7023	6.2219	5.9203	0.13685	0.30164	-15.706	-12.56	-12.596	-10.86	-3.1412	-1.73739	FCC	FCC
PtPdNiSnSb	8.0866	8.0246	6.4183	6.3608	0.06195	0.0575	1.9458	1.9458	-3.696	-3.422	0	-0.27378	BCC	BCC
PtPdScTiV	7.6061	7.5573	6.037	6.032	0.04878	0.00495	-2.0894	-2.199	1.3415	1.4905	0.10997	-0.14905	FCC	FCC
PtPdScTiMn	7.5363	7.5079	5.9816	5.9869	0.02843	-0.00531	-1.6473	-1.83	-0.0201	-0.02	0.18303	-0.00059	FCC	FCC
PtPdScTiZn	7.6531	7.5583	6.0742	6.0718	0.09475	0.00243	-10.555	-8.444	-5.1435	-5.591	-2.1111	0.447264	FCC	FCC
PtPdScTiAl	7.7184	7.7638	6.1261	6.036	-0.04544	0.09007	-8.7906	-8.97	-9.7261	-10.13	0.1794	0.405256	BCC	BCC
PtPdScTiSi	7.8309	7.7006	6.2154	6.0355	0.13033	0.17991	-12.76	-11.81	-11.56	-12.99	-0.9452	1.42879	FCC	BCC
PtPdScTiSb	8.1194	8.0249	6.4444	6.3883	0.09447	0.05605	-3.6006	-3.104	5.904	5.7882	-0.4966	0.115764	FCC	FCC
PtPdScVMn	7.4267	7.4011	5.8946	5.9076	0.02563	-0.013	-1.3237	-1.261	1.4246	1.5318	-0.063	-0.10723	FCC	FCC
PtPdScVZn	7.5468	7.4859	5.9899	5.9933	0.06094	-0.00337	-7.2875	-7.921	-3.9162	-3.435	0.6337	-0.48094	FCC	FCC
PtPdScVAl	7.614	7.6862	6.0432	5.9291	-0.07222	0.11412	-11.06	-9.141	-9.1806	-9.001	-1.9195	-0.18001	FCC	FCC
PtPdScVSi	7.7296	7.6489	6.135	5.9363	0.08068	0.19867	-9.4498	-12.12	-12.908	-11.63	2.66532	-1.27915	BCC	FCC
PtPdScVSb	8.0252	7.965	6.3696	6.3122	0.06022	0.05742	-2.7352	-2.82	5.5631	6.0469	0.08459	-0.48375	FCC	FCC
PtPdScMnZn	7.476	7.4224	5.9337	5.9532	0.05358	-0.01951	-6.0434	-5.925	-6.9339	-7.299	-0.1185	0.36494	BCC	BCC
PtPdScMnAl	7.5444	7.6236	5.988	5.913	-0.07923	0.07497	-8.9332	-8.758	-7.438	-6.701	-0.1752	-0.7371	FCC	FCC
PtPdScMnSi	7.6621	7.5784	6.0814	5.8949	0.08367	0.18649	-9.6745	-10.52	-9.9447	-11.3	0.84126	1.356096	BCC	BCC
PtPdScMnSb	7.9627	7.8278	6.32	6.2607	0.13485	0.05926	-1.9726	-2.076	3.4827	3.4482	0.10382	0.034482	FCC	FCC
PtPdScZnAl	7.6609	7.6113	6.0804	5.972	0.04955	0.10842	-6.5897	-8.786	-9.8106	-10.01	2.19655	0.200216	BCC	BCC
PtPdScZnSi	7.7751	7.5486	6.1711	5.9661	0.22648	0.20498	-10.816	-11.04	-11.263	-11.98	0.22073	0.71892	BCC	BCC
PtPdScZnSb	8.0675	7.8284	6.4031	6.3353	0.23905	0.06784	-1.5885	-1.346	-0.4505	-0.442	-0.2423	-0.00883	FCC	FCC
PtPdScAlSi	7.8384	7.8359	6.2213	5.8734	0.00247	0.34792	-13.125	-12.15	-10.412	-11.97	-0.9722	1.555879	FCC	FCC
PtPdScAlSb	8.1263	8.0683	6.4499	6.3384	0.058	0.11145	-11.281	-9.642	0.1859	0.1999	-1.6391	-0.01399	FCC	FCC
PtPdScSiSb	8.0281	7.9482	6.5306	6.3174	0.07985	0.21321	-14.249	-11.58	-2.2132	-2.088	-2.6644	-0.12527	FCC	FCC
PtPdTiVMn	7.2028	7.2177	5.7169	5.7811	-0.0149	-0.06424	-10.081	-10.61	-7.455	-6.427	0.53056	-1.02827	FCC	FCC
PtPdTiVZn	7.3303	7.3651	5.8181	5.8705	-0.03481	-0.05244	-6.877	-8.931	-9.2226	-9.607	2.05418	0.384276	BCC	BCC

HEAs	Lattice parameters (Bohr)						ΔH_f (kJ/mol*f.u.)						Phase	
	FCC Cal.	FCC ML	BCC Cal.	BCC ML	FCC error	BCC error	FCC Cal.	FCC ML	BCC Cal.	BCC ML	FCC error	BCC error	Cal.	ML
PtPdTiVAl	7.4014	7.594	5.8745	5.7689	-0.1926	0.10559	-8.9912	-9.366	-8.4737	-8.736	0.37463	0.262074	FCC	FCC
PtPdTiVSi	7.5236	7.5556	5.9715	5.8396	-0.03202	0.13187	-11.784	-11.12	-7.1928	-8.268	-0.667	1.074788	FCC	FCC
PtPdTiVSb	7.8347	7.7663	6.2184	6.1847	0.06838	0.03369	-7.7885	-7.636	-8.3874	-8.647	-0.1527	0.259404	BCC	BCC
PtPdTiMnZn	7.2551	7.3214	5.7584	5.8041	-0.06628	-0.04571	-7.9752	-8.764	-12.874	-13.41	0.78876	0.536424	BCC	BCC
PtPdTiMnAl	7.3277	7.5431	5.816	5.7413	-0.21542	0.07468	-6.7819	-8.924	-9.1431	-10.63	2.14164	1.48841	BCC	BCC
PtPdTiMnSi	7.4523	7.4907	5.9149	5.7904	-0.03843	0.12447	-7.8025	-10.13	-10.872	-10.56	2.33061	-0.31666	BCC	BCC
PtPdTiMnSb	7.769	7.6747	6.1663	6.1366	0.09429	0.02965	-4.4285	-5.905	-7.6525	-6.713	1.47615	-0.93978	BCC	BCC
PtPdTiZnAl	7.451	7.5246	5.9138	5.8336	-0.07362	0.08024	-7.5792	-8.613	-13.41	-11.76	1.03352	-1.64679	BCC	BCC
PtPdTiZnSi	7.5716	7.4429	6.0096	5.8756	0.12868	0.13396	-10.031	-10.13	-8.8258	-10.38	0.10132	1.557495	FCC	BCC
PtPdTiZnSb	7.879	7.7151	6.2535	6.2053	0.16387	0.04824	-6.8241	-8.53	-7.4128	-6.864	1.70602	-0.5491	BCC	FCC
PtPdTiAlSi	7.6383	7.8021	6.0625	5.8024	-0.16382	0.26011	-11.436	-11.21	-11.438	-11.21	-0.2242	-0.22427	BCC	BCC
PtPdTiAlSb	7.9406	7.8656	6.3025	6.1342	0.07503	0.16828	-12.441	-11.21	-12.405	-12.41	-1.2329	0	FCC	BCC
PtPdTiSiSb	8.0471	7.8145	6.387	6.1283	0.23261	0.25869	-8.8106	-11.75	-15.107	-15.11	2.93685	0	BCC	BCC
PtPdVMnZn	7.1366	7.2025	5.6644	5.715	-0.06586	-0.05064	-10.28	-9.179	-12.484	-12.61	-1.1015	0.1261	BCC	BCC
PtPdVMnAl	7.2116	7.4651	5.7239	5.6682	-0.2535	0.05565	-8.2612	-9.179	-10.669	-10.07	0.91791	-0.60391	BCC	BCC
PtPdVMnSi	7.3401	7.4412	5.8259	5.7364	-0.10107	0.08946	-8.6738	-10.09	-10.548	-9.951	1.41201	-0.59708	BCC	FCC
PtPdVMnSb	7.666	7.61	6.0845	6.0587	0.05599	0.0258	-6.168	-6.047	-7.8146	-7.169	-0.1209	-0.64525	BCC	BCC
PtPdVZnAl	7.3388	7.4622	5.8248	5.7716	-0.12341	0.0532	-11.084	-9.011	-11.94	-11.71	-2.0726	-0.23412	BCC	BCC
PtPdVZnSi	7.463	7.3815	5.9234	5.8217	0.08151	0.1017	-9.3172	-10.24	-10.332	-10.76	0.92148	0.430516	BCC	BCC
PtPdVZnSb	7.7789	7.6404	6.1741	6.129	0.13848	0.0451	-7.8028	-7.576	-8.8303	-8.331	-0.2273	-0.49983	BCC	BCC
PtPdVAlSi	7.5317	7.8007	5.9779	5.7555	-0.26905	0.22237	-8.9187	-11.01	-11.52	-10.19	2.09205	-1.32532	BCC	FCC
PtPdVAlSb	7.8421	7.8174	6.2243	6.0467	0.02472	0.17759	-13.002	-11.02	-13.044	-12.19	-1.9834	-0.85331	BCC	BCC
PtPdVSiSb	7.9512	7.7763	6.3109	6.0403	0.17493	0.2706	-9.9179	-11.81	-17.153	-15.45	1.88912	-1.6999	BCC	BCC
PtPdMnZnAl	7.2638	7.3895	5.7653	5.7128	-0.12571	0.05248	-7.1796	-8.447	-14.238	-12.38	1.26699	-1.85715	BCC	BCC
PtPdMnZnSi	7.3905	7.3196	5.8659	5.7743	0.07093	0.09157	-10.252	-9.236	-11.191	-13.48	-1.016	2.292042	BCC	BCC
PtPdMnZnSb	7.7122	7.6372	6.1212	6.0685	0.07504	0.05271	-5.2222	-5.739	-7.9486	-8.456	0.51648	0.50736	BCC	BCC
PtPdMnAlSi	7.4605	7.7344	5.9214	5.7442	-0.27391	0.1772	-12.148	-9.877	-13.741	-13.34	-2.2716	-0.40023	BCC	BCC
PtPdMnAlSb	7.7766	7.7524	6.1723	6.034	0.02416	0.13826	-8.7308	-10.04	-9.1064	-9.792	1.3046	0.685426	BCC	FCC

HEAs	Lattice parameters (Bohr)						ΔH_f (kJ/mol*f.u.)						Phase	
	FCC Cal.	FCC ML	BCC Cal.	BCC ML	FCC error	BCC error	FCC Cal.	FCC ML	BCC Cal.	BCC ML	FCC error	BCC error	Cal.	ML
PtPdMnSiSb	7.8875	7.724	6.2603	6.0183	0.16348	0.242	-7.9097	-10.55	-19.017	-18.46	2.63655	-0.5539	BCC	BCC
PtPdZnAlSi	7.5795	7.6425	6.0159	5.8116	-0.06296	0.20429	-10.928	-9.671	-14.593	-15.04	-1.2572	0.451326	BCC	BCC
PtPdZnAlSb	7.8863	7.7219	6.2594	6.0786	0.16443	0.18078	-8.9444	-9.829	-9.5321	-9.827	0.88461	0.294807	BCC	FCC
PtPdZnSiSb	7.9943	7.7248	6.345	6.0735	0.26945	0.27154	-10.051	-9.951	-19.786	-18.32	-0.0995	-1.46562	BCC	BCC
PtPdAlSiSb	8.0542	8.0076	5.9259	5.9475	0.04656	-0.0216	-9.6707	-10.87	-22.958	-20.5	1.19525	-2.45974	BCC	BCC

References

1. Troparevsky, M.C., et al., *Beyond atomic sizes and Hume-Rothery rules: understanding and predicting high-entropy alloys.* Jom, 2015. **67**(10): p. 2350-2363.
2. Hu, Y., et al., *First-principle calculation investigation of NbMoTaW based refractory high entropy alloys.* Journal of Alloys and Compounds, 2020. **827**: p. 153963.
3. Akai, H., *Fast Korringa-Kohn-Rostoker coherent potential approximation and its application to FCC Ni-Fe systems.* Journal of Physics: Condensed Matter, 1989. **1**(43): p. 8045.
4. Akai, H. *AkaiKKR (MACHIKANEYAMA)*. Available from: http://kkr.issp.u-tokyo.ac.jp.
5. Huang, E.-W., et al., *Machine-learning and high-throughput studies for high-entropy materials.* Materials Science and Engineering: R: Reports, 2022. **147**: p. 100645.
6. Lucas, M., et al., *Absence of long-range chemical ordering in equimolar FeCoCrNi.* Applied Physics Letters, 2012. **100**(25): p. 251907.
7. Zhang, Y., X. Yang, and P. Liaw, *Alloy design and properties optimization of high-entropy alloys.* Jom, 2012. **64**(7): p. 830-838.
8. Lucas, M., et al., *Magnetic and vibrational properties of high-entropy alloys.* Journal of Applied Physics, 2011. **109**(7): p. 07E307.
9. Wu, Z., et al., *Recovery, recrystallization, grain growth and phase stability of a family of FCC-structured multi-component equiatomic solid solution alloys.* Intermetallics, 2014. **46**: p. 131-140.
10. Cantor, B., et al., *Microstructural development in equiatomic multi-component alloys.* Materials Science and Engineering: A, 2004. **375**: p. 213-218.
11. Senkov, O., et al., *Refractory high-entropy alloys.* Intermetallics, 2010. **18**(9): p. 1758-1765.
12. Senkov, O.N., et al., *Mechanical properties of Nb25Mo25Ta25W25 and V20Nb20Mo20Ta20W20 refractory high entropy alloys.* Intermetallics, 2011. **19**(5): p. 698-706.
13. Yeh, J.-W., et al., *Formation of simple crystal structures in Cu-Co-Ni-Cr-Al-Fe-Ti-V alloys with multiprincipal metallic elements.* Metallurgical and Materials Transactions A, 2004. **35**(8): p. 2533-2536.
14. Senkov, O., et al., *Microstructure and elevated temperature properties of a refractory TaNbHfZrTi alloy.* Journal of Materials Science, 2012. **47**(9): p. 4062-4074.
